# Forecasting brain activity based on models of spatiotemporal brain dynamics: A comparison of graph neural network architectures

**DOI:** 10.1162/netn_a_00252

**Published:** 2022-07-01

**Authors:** S. Wein, A. Schüller, A. M. Tomé, W. M. Malloni, M. W. Greenlee, E. W. Lang

**Affiliations:** CIML, Biophysics, University of Regensburg, Regensburg, Germany; Experimental Psychology, University of Regensburg, Regensburg, Germany; IEETA, DETI, Universidade de Aveiro, Aveiro, Portugal

**Keywords:** Brain connectivity, Graph neural networks, Structure-function relationship, Directed connectivity

## Abstract

Comprehending the interplay between spatial and temporal characteristics of neural dynamics can contribute to our understanding of information processing in the human brain. Graph neural networks (GNNs) provide a new possibility to interpret graph-structured signals like those observed in complex brain networks. In our study we compare different spatiotemporal GNN architectures and study their ability to model neural activity distributions obtained in functional MRI (fMRI) studies. We evaluate the performance of the GNN models on a variety of scenarios in MRI studies and also compare it to a VAR model, which is currently often used for directed functional connectivity analysis. We show that by learning localized functional interactions on the anatomical substrate, GNN-based approaches are able to robustly scale to large network studies, even when available data are scarce. By including anatomical connectivity as the physical substrate for information propagation, such GNNs also provide a multimodal perspective on directed connectivity analysis, offering a novel possibility to investigate the spatiotemporal dynamics in brain networks.

## Author Summary

In our study we compare different spatial and temporal modeling techniques based on graph neural networks (GNNs) for investigating the spatiotemporal dynamics in brain networks. We show that a convolutional neural network and a recurrent neural network–based approach are both very suitable to capture the temporal characteristics in functional activity distributions. Further, we demonstrate that structural connectome embeddings can effectively reduce the number of parameters in GNN models, by naturally including higher order topological relations between brain areas within the structural network. We compare the prediction accuracy of the GNN-based approaches to a vector autoregressive model, and we show that GNNs remain considerably more accurate when brain networks become large and available data are limited. Finally, we demonstrate how these spatiotemporal GNN models can provide a multimodal perspective on directed connectivity in brain networks.

## INTRODUCTION

Distinct concepts of brain connectivity can provide different but complementary aspects of information processing in the brain (Lang, Tomé, Keck, Górriz-Sáez, & Puntonet, 2012). On the one hand, imaging modalities like functional magnetic resonance imaging (fMRI) allow us to temporally resolve dynamic neural activity patterns in distinct spatial locations in the human brain. Different statistical approaches that describe the coherency of activity profiles in brain networks have been proposed based on the notion of functional connectivity (FC). On the other hand, diffusion tensor imaging (DTI) can provide insights into the structural and relatively static aspects of the brain. By reconstructing white matter tracks from DTI data, the anatomical or structural connectivity (SC) between different brain areas can be estimated. Also, directed and potentially causal relationships between regions become of interest in fMRI and are studied with respect to directed functional or effective connectivity. The latter is most often inferred from Granger causality or dynamic causal modeling (Bielczyk et al., 2019; Friston, Moran, & Seth, 2013).

Based on these concepts, spatial and temporal relationships between brain areas can be represented by graphical models, which have recently received increasing attention in the field of machine learning (Bronstein, Bruna, LeCun, Szlam, & Vandergheynst, 2017; Wu et al., 2021). So-called graph neural networks (GNNs) allow us to effectively process signals in the non-Euclidean geometry of graphs, providing also novel possibilities for applications in brain connectivity research (Arslan, Ktena, Glocker, & Rueckert, 2018; Kim & Ye, 2020; Ktena et al., 2018; X. Li et al., 2019; Rosenthal et al., 2018; Wein, Malloni, et al., 2021). Given a decomposition of the brain into specified areas, their spatiotemporal neural activity patterns can be interpreted as graph-structured signal distributions. Nodes in brain networks can be associated with variables like the temporal neuronal activity of neuron pools, while edges in such networks reflect the strength of interactions between neural populations (Bullmore & Bassett, 2011). As proposed in our recent study (Wein, Malloni, et al., 2021), these complex signals exhibited in non-Euclidean geometries can be processed with a variant of GNN denoted as spatiotemporal graph neural network (STGNN). Such STGNNs can allow us to simultaneously model spatial and temporal dependencies in such graph structured signals and thereby provide a new possibility to combine DTI with fMRI data. Activity propagation–based approaches made already various interesting contributions to brain connectivity research, and could, for example, explain how resting-state FC (Cole, Ito, Bassett, & Schultz, 2016) or SC (Yan et al., 2021) are related to cognitive task activations observed in task-based fMRI. Moreover, they could give us insights into what way the hierarchical organization of the brain is related to the propagation of information along structural connections (Vézquez-Rodríguez, Liu, Hagmann, & Misic, 2020). In our study, we compare different approaches based on GNNs to study the spatiotemporal propagation of information in brain networks from a multimodal and data-driven perspective.

Recently, several different GNN architectures have been proposed to model the flow of information across time and space in graphical signals (Wu et al., 2021). Convolution operations, often applied in deep learning, have recently been extended successfully to graphical models and allow us to capture inherent spatial dependencies on non-Euclidean network structures (Defferrard, Bresson, & Vandergheynst, 2016). They were later combined with recurrent neural networks (RNNs) (Rumelhart, Hinton, & Williams, 1986), which can detect sequential relations in signals. This combined spatiotemporal GNN framework was proposed in the notion of diffusion convolution recurrent neural network (DCRNN) (Y. Li, Yu, Shahabi, & Liu, 2018). However, RNNs can have problems with long time series and, when combined with graph convolution operations, their gradients are more likely to explode (exploding gradients) or vanish (vanishing gradients) (Y. Li et al., 2018; Seo, Defferrard, Vandergheynst, & Bresson, 2018). This was the motivation for introducing approaches that combine spatial graph convolutions with standard one-dimensional temporal convolutions (Wu et al., 2021). But their receptive field size can only grow if many hidden layers are used (linear growth) or global pooling is applied. To alleviate such shortcomings, so-called WaveNets (WNs) have been introduced that employ stacked dilated temporal convolutions, which provide a long-term temporal memory (van den Oord et al., 2016). They have recently been combined with graph convolution operations in an architecture denoted as graph WaveNet (GWN) (Wu, Pan, Long, Jiang, & Zhang, 2019). As an alternative method for the temporal processing, also attention mechanisms have been recently included in STGNN architectures (Zheng, Fan, Wang, & Qi, 2020). Attention mechanisms select, from all inputs, information that is critical to the task at hand and modify edge connection strengths accordingly. They have been applied already to natural language processing, speech recognition, and image processing (J. Liang et al., 2019; Vaswani et al., 2017; K. Xu et al., 2015), but applications to analyze the dynamics in neural signals are still missing. In this study we compare these different STGNN architectures with each other and evaluate their effectiveness in replicating functional activity distributions observed in brain networks. In addition to these distinct temporal models, we study different approaches to model the spatial information exchange between brain regions. At first we employ the structural connectivity as the substrate for information propagation between brain regions. Further, we evaluate the effectiveness of employing connectome embeddings (CEs) of the anatomical network to characterize the node relations. In a recent study, Rosenthal et al. (2018) have shown that embeddings of nodes in the anatomical network can inherently capture higher order topological relations between different structurally connected nodes in this network. Finally, we compare it to the case when we incorporate no predefined spatial layout into the GNN models, trying to learn the spatial structure by gradient descent–based optimization during model training. We demonstrate that by modeling the functional information exchange between regions in STGNNs based on structural connectivity, we can significantly increase the accuracy of predicting future neural signals. This points out that STGNN models are capable of learning informative functional interactions between areas in such brain networks. Based on these different comparisons, we at first try to identify the most effective STGNN architectures to investigate such spatial and temporal dynamics in brain networks.

In a subsequent step we then compare this STGNN-based approach to a currently popular data-driven model for characterizing directed functional information exchange in brain networks. Until now, methods for the inference of directed functional or effective connectivity are often based on Granger causality (Barnett & Seth, 2013) or dynamic causal modeling (Friston et al., 2013) and its recent extensions (Frässle et al., 2018; Prando et al., 2020). In addition various algorithmic and information theory–based methods have been developed meanwhile in this field (Bielczyk et al., 2019; Ramsey, Hanson, & Glymour, 2011). In the following, we compare the performance of the STGNN prediction models to the forecasting model most often used in Granger causality analysis (Barnett & Seth, 2013). Granger causality is based on the idea that if one event *A* would cause another event *B*, then *A* should precede *B* and the occurrence of event *A* should contain information about the occurrence of event *B* (Friston et al., 2013). In the context of neuroimaging, this is realized in a predictive framework, by testing if adding information on activity in a region *A* improves the prediction of activity in region *B*. For practical applications of this idea, the underlying predictive model in Granger causality is usually based on a vector autoregression (VAR) for multivariate time series inference (Barnett & Seth, 2013; Bielczyk et al., 2019; Friston et al., 2013). In a brain network with *N* regions of interest (ROIs), the parameters in a VAR model grow with *N*^2^, so for larger brain networks it can be challenging to accurately fit the model if only limited data are available. This can be problematic in fMRI, where the temporal sampling rate is relatively low, while its good spatial resolution would allow for a detailed, high-resolution network analysis. Therefore it would be desirable to have a predictive model that can learn interactions between all brain areas of interest and, in addition, naturally scales to larger brain networks. In our study we compare the STGNN approaches to a classical VAR model and test their accuracy on a variety of network sizes and data set sizes. We show that by learning localized functional interactions based on the anatomical network, the prediction accuracies of STGNN models remain significantly more accurate, even when brain networks become very complex and only limited data are available to fit the models. This points out that the STGNN approaches are robust among a large variety of MRI study scenarios, and are therefore also suitable for the analysis of smaller subject cohorts, like in studies of patients with rare neurological diseases.

Finally, we analyze the spatial interactions between brain regions, which are learned by the STGNN models. By integrating prior knowledge on the brain anatomy in form of structural connectivity or based on connectome embeddings, such models can provide multimodal perspective on directed relations between brain areas. So far, a variety of approaches were proposed to study the structure-function relation in the human brain (Suárez, Markello, Betzel, & Misic, 2020), which are based on computational modeling (Chen & Wang, 2018; Deco et al., 2013; Deco, Senden, & Jirsa, 2012; Honey et al., 2009; Messé, Hütt, König, & Hilgetag, 2015; Messé, Rudrauf, Benali, & Marrelec, 2014), graph theory (Abdelnour, Dayan, Devinsky, Thesen, & Raj, 2018; Becker et al., 2018; H. Liang & Wang, 2017; Lim, Radicchi, van den Heuvel, & Sporns, 2019), and machine learning (Amico & Goñi, 2018; Deligianni, Carmichael, Zhang, Clark, & Clayden, 2016; Rosenthal et al., 2018; Sarwar, Tian, Yeo, Ramamohanarao, & Zalesky, 2021). Biophysically inspired models, for example, could describe how functional connectivity patterns emerge from neural dynamics with static couplings characterized by anatomical connections (Deco et al., 2013; Honey et al., 2009; Messé et al., 2014), and were recently used to also study spatial heterogeneity of local circuit properties across the cortex (Demirtaş et al., 2019; P. Wang et al., 2019). Methods from graph theory can supplement such computational frameworks by specifically pointing out how indirect structural connections contribute to the inference of FC strength (Becker et al., 2018; H. Liang & Wang, 2017). Also, hybrid methods could demonstrate that the typology of structural brain networks supports in neuromorphic networks their memory capacity (Suárez, Richards, Lajoie, & Misic, 2021). Such insights into structural and functional connectivity can then provide a basis to better understand the cognitive information processing in the human brain (Ito, Hearne, Mill, Cocuzza, & Cole, 2020). The vast majority of studies on structure-function relations focuses currently on inferring overall FC patterns from their SC, although static coherency–based measures of FC might have limitations in their ability to capture the rich nature of dynamic brain activity (Wein, Deco, et al., 2021). To the contrary, STGNNs are able to directly predict the measured BOLD dynamics, and their interactions between brain regions, without relying on the indirect representation of functional dynamics based on coherency. This characteristic of STGNNs allows us to additionally investigate the structure-function coupling in brain networks from a novel perspective. To study this relation further on the individual brain region level, we demonstrate how a perturbation-based approach can be utilized to reconstruct how dynamic functional interactions are mediated by their structural substrate in STGNN models. By inferring how information is propagated between individual regions in STGNNs, these predictive models have the potential to reveal directed relationships between individual areas in brain networks from a multimodal perspective. In general, due to the low temporal sampling rate and physiological artifacts, fMRI can have several limitations in detecting directed relations in brain networks (S. M. Smith et al., 2011; Webb, Ferguson, Nielsen, & Anderson, 2013). Still, some recent fMRI studies and computational simulations could demonstrate that also lag-based methods like Granger causality can be useful for detecting such directed dependencies in fMRI data (Duggento et al., 2018; Mill, Bagic, Bostan, Schneider, & Cole, 2017; Seth, Chorley, & Barnett, 2012; H. E. Wang et al., 2014).

The possibility to combine structural and functional imaging data in STGNNs can make these models as well interesting for several practical applications in brain connectivity research. For instance, they can be used to investigate differences in the structure-function relationship between resting-state and task-based fMRI. Furthermore, in clinical applications these models could be employed to study how lesions in the structural connectome affect the functional organization of the brain network. In our current study we compare, therefore, such mechanisms for spatial and temporal modeling in STGNNs, with the objective to establish their methological foundation for brain connectivity research, and thereby providing a basis for future applications of STGNNs in multimodal neuroimaging studies.

## RESULTS

### Graph Neural Network Models

**Figure 1.**
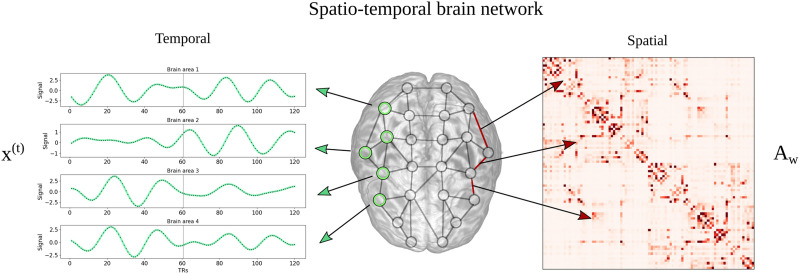
The spatiotemporal representation of a signal in a brain network is illustrated. The temporal component of the graph-like signal is given by the BOLD signal **x**^(*t*)^ ∈ ℝ^*N*^ in *N* brain regions sampled at different timesteps *t*, as shown on the left side. The strength of the edges in the brain network are defined by a weighted adjacency matrix **A**_*w*_ ∈ ℝ^*N*×*N*^, as illustrated on the right side. One entry *w*_*nn*′_ of the matrix **A**_*w*_ characterizes thereby the spatial relation between brain region *n* and *n*′ in the network.

In our context of MRI, the goal of the spatiotemporal GNNs will be to forecast the observed BOLD signal as accurately as possible in order to precisely capture the spatiotemporal dynamics of the underlying mechanisms in the brain. The learning objective can be formalized by introducing a graph signal **x**^(*t*)^ ∈ ℝ^*N*^, representing the BOLD signal measured at time step *t* in *N* different brain regions. The goal of the models is to predict from an input sequence of *T*_*p*_ past neural activity states *t* = 1, …, *T*_*p*_ a sequences of future states *t* = 1, …, *T*_*f*_. In addition to the temporal information, also spatial dependencies are included in the GNN architectures. The spatial relations between the *N* brain regions can be represented in the notion of a graph 𝒢 = (𝒱, 𝓔, **A**_*w*_), containing vertices (nodes) 𝒱, with |𝒱| = *N*, and edges 𝓔. The structure of the graph is characterized by a weighted adjacency matrix **A**_*w*_ ∈ ℝ^*N*×*N*^, where an entry *w*_*nn*′_ describes the connection strength between brain region *n* and *n*′. An illustration of such a graphical representation of a dynamic brain state is provided in Figure 1. Based on this concept, the task of the GNN models is to derive a function *h*(·) that best predicts *T*_*f*_ future activity states from an input sequence of *T*_*p*_ past states:

$$\left[x^{\left(1\right)},\ldots ,x^{\left(T_{p}\right)};𝒢\right]\overset{h\left(\cdot \right)}{\to }\left[x^{\left(T_{p}+1\right)},\ldots ,x^{\left(T_{p}+T_{f}\right)}\right]$$ (1)

**Figure 2.**
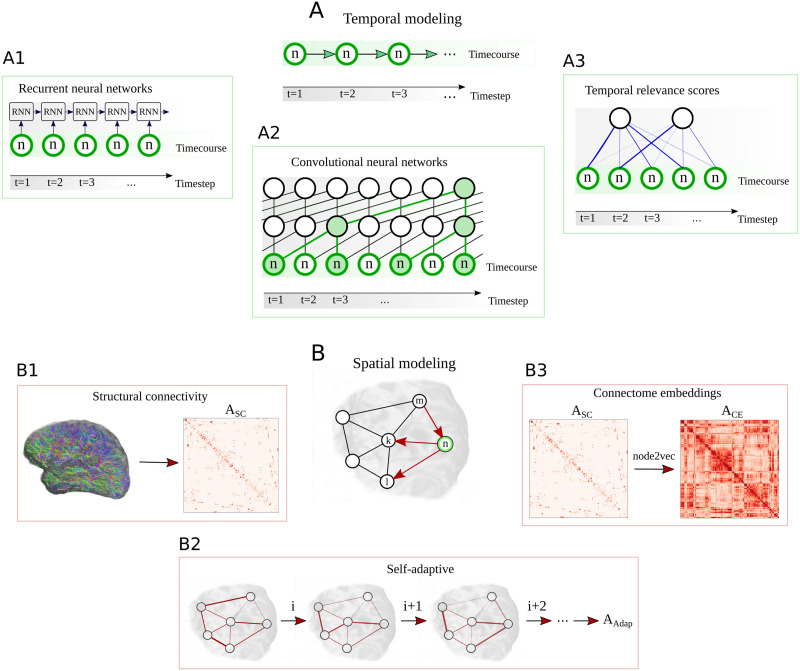
Overview on the different techniques used by spatiotemporal GNNs to learn the spatial and temporal dynamics in brain networks. The temporal component of the STGNN tries to infer dependencies between the activity at different timesteps *t* = 1, 2, 3, … in a certain brain region *n*, as illustrated in A. A recurrent neural network (RNN) architecture processes in a recursive manner the activity states at the different time steps *t* (A1). In our study, this principle will be implemented in an RNN-based sequence-to-sequence architecture for time series forecasting. As an alternative, convolutional neural network (CNN) architectures employ one-dimensional convolutions in the time domain (A2). This idea is picked up in the WaveNet (WN) architecture, which introduces dilated convolutions to obtain exponentially growing receptive fields, as highlighted here in green. Following the idea of an attention mechanism, temporal relevance scores can be dynamically computed to weight the importance of a temporal feature observed at time step *t* (A3). A temporal attention (TAtt) based architecture is thereby composed of a multitude of stacked attention layers. Based on these temporal modeling approaches, STGNNs additionally detect spatial dependencies between a node *n* and other interconnected nodes in the brain network, as illustrated in B. The substrate for the spatial interactions can be based on the structural connectivity matrix *A*_*SC*_, as reconstructed from DTI data (B1). Alternatively, the node2vec algorithm can be used to detect higher order relationships between brain regions in the structural connectome, for characterizing the spatial similarity based on connectome embeddings *A*_*CE*_ (B3). Finally, spatial connections can be tried to be learned by the model itself, by freely adapting the edges in an adjacency matrix *A*_*Adap*_ at different iterations *i*, *i* + 1, *i* + 1 … during the model training (B2).

Until now various spatiotemporal GNN architectures have been proposed to account for spatial and temporal dependencies of such graph structured signals (Wu et al., 2021). An overview of different possibilities for the temporal modeling is given in Figure 2A. In time series analysis, recurrent neural networks (RNNs) (Rumelhart et al., 1986) provide one efficient way to detect patterns in sequential data structures, like in our context the BOLD signal, subsequently sampled at different time steps *t* (Figure 2A1). This approach can be extended to a RNN-based sequence-to-sequence architecture, whereby an encoder recursively processes an input sequence of *T*_*p*_ past neural activity states **x**^(*t*)^ and encodes the temporal information into a hidden state **H**^(*T*_*p*_)^ (Sutskever, Vinyals, & Le, 2014). Next, a decoder network uses the information in **H**^(*T*_*p*_)^ to generate a prediction for *T*_*f*_ future activity states. To account for vanishing gradients during training, the encoder and decoder consist of gated recurrent unit (GRU) cells (Chung, Gulcehre, Cho, & Bengio, 2014). An alternative for detecting repetitive patterns in sequential data is provided by convolutional neural networks (CNNs) (Figure 2A2). By employing one-dimensional convolutions in the time domain, they are used in our context to process temporal dynamics of neural activity. To more efficiently capture long-term dependencies in temporal data the WaveNet (WN) architecture has been proposed (van den Oord et al., 2016). This model introduces dilated causal convolution operations to generate a large receptive field when using only relatively few network layers, which alleviates the processing of long temporal input horizons. The growth of the receptive field of a neuron (marked in green) in a WN layer is illustrated in Figure 2A2. More recently, also attention mechanisms have been proposed to detect underlying hidden correlations in sequential data structures (Vaswani et al., 2017). In time series analysis, the idea of a temporal attention (TAtt) architecture is thereby to adaptively focus on the most important temporal features in a sequence (Figure 2A3).

These different fundamental approaches for temporal dependency modeling have been recently combined with techniques to additionally capture spatial relationships in graph structured signals (Y. Li et al., 2018; Wu et al., 2019; Zheng et al., 2020). Graph convolutional neural networks can be incorporated to model the propagation of information between adjacent nodes in the graphical representation of the signal (Defferrard et al., 2016). The neighborhoods of the vertices/nodes 𝒱 in the network are characterized by the adjacency matrix **A**_*w*_. In our study we additionally investigate different possibilities for defining the spatial layout for the information propagation between brain regions, as illustrated in Figure 2B. As a first choice for the adjacency matrix, we will employ the structural connectivity **A**_*SC*_ between the *N* brain areas, as it could be reconstructed from DTI data (Figure 2B1). This choice is motivated by the idea that white matter connections obtained from this modality would establish the anatomical backbone for information exchange between brain areas. In a recent study, Rosenthal et al. (2018) demonstrated that connectome embeddings (CE) can be utilized for projecting the structural connectome into a continuous vector space, which captures meaningful correspondences between different brain areas. This technique can thereby allow us to additionally account for long-range and interhemispheric homotopic connections, which are usually only weakly expressed in DTI-based anatomical connectivity (Thomas et al., 2014). In our study, we utilized this technique to represent the edge weight *w*_*nn*′_ in the adjacency matrix as the similarity between the vector representations of two nodes *n* and *n*′, which will be denoted as **A**_*CE*_ (Figure 2B3). The information is accordingly propagated between brain regions that possess high similarity based on their neighborhood role within the anatomical network. Finally, we compare these techniques to the case when the model is given the freedom to learn spatial dependencies between the *N* regions itself. In this setup, the adjacency matrix is represented by a self-adaptive matrix **A**_*Adap*_ ∈ ℝ^*N*×*N*^, which is learned during the model optimization (Figure 2B2). A detailed formal description of the model architectures and the training involved is outlined in the Materials and Methods section. In the following, we will assess the effectiveness of the different spatial and temporal modeling approaches by comparing their predictive performance on an MRI dataset from the Human Connectome Project (HCP) (Van Essen et al., 2013).

### Data Description

For the different evaluations in this study, resting-state fMRI data provided by the HCP *S1200 release* was incorporated (S. M. Smith et al., 2013). To define the nodes of the brain network, the multimodal parcellation proposed by Glasser et al. (2016) was applied, which is composed of 180 segregated regions within each hemisphere. The average of the BOLD signal was computed within each brain region, so for each resting-state session, *N* = 360 time courses were obtained (180 per hemisphere). During one session, *T* = 1,200 fMRI images were collected, so that the ROI time series can be represented by a data matrix **X** ∈ ℝ^*N*×*T*^. We filtered the resting-state fMRI time series data with a 0.04–0.07 Hz Butterworth band-pass filter, because this frequency band has shown to be most reliable and functionally relevant for gray matter activity (Achard, Salvador, Whitcher, Suckling, & Bullmore, 2006; B. B. Biswal, Yetkin, Haughton, & Hyde, 1995; Buckner et al., 2009; Deco, Kringelbach, Jirsa, & Ritter, 2017; Glerean, Salmi, Lahnakoski, Jääskeläinen, & Sams, 2012).

For learning the predictions of the BOLD signal, samples of input and output sequences were generated from the time series data in **X** (Wein, Malloni, et al., 2021). This was achieved by selecting windows of length *T*_*p*_ to obtain input sequences of neural activity states [**x**^(1)^, …, **x**^(*T*_*p*_)^], and respective target sequences of length *T*_*f*_ denoted as [**x**^(*T*_*p*_+1)^, …, **x**^(*T*_*p*_+*T*_*f*_)^]. The time index *t* was propagated through each fMRI session, where in total *T* − *T*_*p*_ − *T*_*f*_ + 1 input-output pairs were generated per session. For each fMRI session, the first 80% of those time window samples were used as the training data for the models, the subsequent 10% as a validation set, and the last 10% were employed for testing. For the following comparisons, the length of the input and output sequences were selected to be *T*_*p*_ = *T*_*f*_ = 60, which corresponds to a time span of roughly 43 s, based on a sampling interval of *TR* = 0.72 s (Uğurbil et al., 2013). This time window has been shown to be long enough to be sufficiently challenging for the models and to make clear the differences in their performance. Likewise, the time window of 60 time points is short enough for them to make reasonable nonrandom forecasts of the BOLD signal.

In addition to the functional dynamics in the different brain regions derived from fMRI, the structural connectivity between those regions was reconstructed from DTI data. For this purpose, the DTI dataset in the HCP *S1200 release* was processed using the multishell, multitissue constrained spherical deconvolution model (Jeurissen, Tournier, Dhollander, Connelly, & Sijbers, 2014), made available in the *MRtrix3* software package (Tournier et al., 2019). White matter tractography was performed to estimate the anatomical connection strength between the regions defined by the multimodal parcellation atlas (Glasser et al., 2016). The number of the streamlines that connect two atlas regions was used to determine the structural connectivity values between the *N* brain regions, which were then collected in a structural connectivity matrix **A**_*SC*_ ∈ ℝ^*N*×*N*^. A detailed description of the MRI datasets and their preprocessing is provided in the Dataset section. In addition, the embeddings of the nodes within the structural network **A**_*SC*_ ∈ ℝ^*N*×*N*^ were generated using the node2vec algorithm (Grover & Leskovec, 2016). The parameters for this algorithm are outlined in detail in the Spatial Dependencies section, and further Pearson correlation was used to quantify the degree of similarity of structural nodes in their connectome embedding space. The pairwise similarities between the *N* nodes were then collected in the matrix **A**_*CE*_ ∈ ℝ^*N*×*N*^.

### Comparison of GNN Architectures

**Figure 3.**
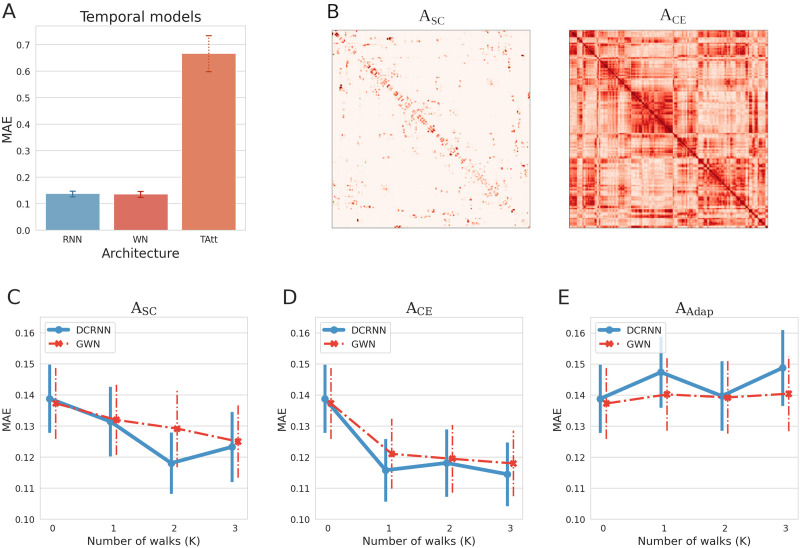
(A) A comparison of different modeling strategies for temporal dynamics in the BOLD signal, comparing the test MAE of the recurrent neural network (RNN), the WaveNet (WN), and the temporal attention (TAtt) architecture. The overall test error was computed as an average across samples, brain regions, and subject sessions. The error bars represent the standard deviation of the test MAE across subjects. Due to their high accuracy in the temporal domain, we focus on RNN- and WN-based approaches for forecasting the spatiotemporal dynamics in the following. Spatial relations are added to the temporal models in form of graph convolutions, and the spatiotemporal extension of the RNN and WN models are respectively denoted as diffusion convolution recurrent neural network (DCRNN) and graph WaveNet (GWN) (Y. Li et al., 2018; Wu et al., 2021). Spatial transitions are based on the relations of network nodes captured in a weighted adjacency matrix, which is either based on structural connectivity (**A**_*SC*_), connectome embedding similiarity (**A**_*CE*_), or adapted during model training (**A**_*Adap*_). In (B) the adjacency matrix **A**_*SC*_ based on structural connectivity within the 180 regions of the right hemisphere is illustrated, together with the adjacency matrix **A**_*CE*_ derived from structural connectome embedding similarities. The regions in this illustration are ordered according to the atlas proposed by Glasser et al. (2016). (C, D, and E) Prediction accuracies of the DCRNN and GWN model in dependence of the walk order *K*. In (C) the overall test MAE is shown when incorporating the SC as an adjacency matrix **A**_*SC*_, (D) illustrates the test MAE when employing CEs in an adjacency matrix **A**_*CE*_ to define spatial relationships, and (E) displays the case when using a self-adaptive weight matrix **A**_*Adap*_.

Before evaluating the performance of different models on a larger variety of MRI study scenarios, we will first focus on the effects of different temporal and spatial modeling techniques. For this purpose, a dataset with a sample size of a medium-sized fMRI study including 25 subjects will be incorporated. Each resting-state fMRI session was decomposed into pairs of input and output samples, as discussed in the Data Description section, and the generated training, validation, and test samples were then aggregated across the 25 fMRI sessions. The neural signal in regions within the right hemisphere (Glasser et al., 2013), consisting of *N* = 180 ROIs, will be included in the following comparison. At first we evaluate the prediction accuracy of the different temporal modeling strategies. For this purpose, we compare the recurrent neural network (RNN) model, with the WaveNet (WN) model and the temporal attention (TAtt) model. The influence of the model hyperparameters, which are used in the following comparisons, are described in the Model Training section and discussed in detail in Supporting Information I. The BOLD signal data was scaled to zero mean and unit variance for the evaluations, to obtain values of a magnitude that are easier to interpret. Figure 3A shows the test mean absolute error (MAE) between the predicted and the true neural activity. By generating windowed input-output pairs of activity values from the fMRI data, the last 10% of samples from each session correspond to 108 of such input-output pairs per session for testing, each containing 60 output time points (corresponding to roughly 43 s of activity). The overall test errors were computed as the average across all these test samples from the 25 subjects and across all 180 brain regions. The comparison shows that RNN and WN have very similar capabilities in predicting the BOLD signal, while the TAtt model exhibits a worse performance. To test the significance of this difference between the models, we further computed the test MAE of each individual subject as an average across predicted time points, brain regions, and test samples per subject. By applying a paired *t* test, the differences between the WN and RNN model to the TAtt model were shown to be both highly significant with *p* ≤ 0.0001 across subjects (Cohen’s *d* = 10.68 and *d* = 10.66, respectively). In addition to the MAE, we evaluated these models in Supporting Information III using scale-free measures like R-squared (*R*^2^) and the similarity of the predicted FC states. Despite their conceptual differences, the results show that the RNN- and WN-based approach can both recover a comparable and consistent amount of temporal information from the fMRI data. In comparison to these, the TAtt architecture appears to be less suitable to accurately predict the BOLD signal with this limited amount of data. For this reason, in the following we will focus on RNN- and WN-based approaches for identifying suitable models to model functional dynamics in brain networks.

In the next step, we will study the impact of adding information on spatial relations between the different regions in the brain network. This will be implemented by invoking graph convolution operations in the predictive models, as outlined in detail in the Spatial Dependencies section. The definition of an adjacency matrix determines how information is propagated between the different nodes in our brain network, and in our evaluations we investigate three conceptually different possibilities. In the first approach we use the structural connectivity as derived from DTI as the substrate for information exchange between different ROIs. The SC-based adjacency matrix **A**_*SC*_ is illustrated in Figure 3B. The information can propagate along direct connections in the graph (*K* = 1), but also higher orders (*K* = 2, 3, …) expressing the influence of indirect connections can considerably contribute to interactions between different areas in the brain (Becker et al., 2018; Bettinardi et al., 2018; H. Liang & Wang, 2017). A walk order of *K* = 0 denotes the case when including no spatial information exchange between network areas, exclusively incorporating temporal information for the predictions. Figure 3C depicts the test MAE in dependence of the walk order *K* when using the SC derived from DTI as a basis for information propagation in space. The RNN-based model in combination with graph convolution operations is referred to as DCRNN (Y. Li et al., 2018) and the MAE of its predictions, averaged across test samples, brain regions, and predicted time points is depicted in blue. Figure 3C shows that it has the lowest test MAE when incorporating walks on the structural graph up to a order of *K* = 2. The WN incorporating graph convolution operations is denoted as GWN (Wu et al., 2019), and its average test MAE is shown in red in Figure 3C. The influence of the walk order *K* on the GWN accuracy suggests that its performance can be successively improved by including first-order connections, followed by the second- and third-order connections. As an alternative thereto, the structural similarity between ROIs can be based on their CE similiarity **A**_*CE*_, as illustrated in Figure 3B. The comparison between **A**_*CE*_ and the structural connectivity matrix **A**_*SC*_ highlights that in the adjacency relation defined by the structural embeddings, long-range connections between brain regions are considerably more pronounced. Figure 3D shows the test MAE of the models when incorporating **A**_*CE*_ in the graph convolution operations. In this case we can observe for both models a sharp drop in the error at walk order *K* = 1. This suggests that the node embeddings already inherently capture higher order relations between nodes in the brain network. Finally, in Figure 3E the test MAE is shown when treating the connections between nodes as learnable weights. In this case, we do not observe an improvement in the test error. This observation indicates that it is rather challenging to learn all *N*^2^ connections between brain regions without any prior knowledge. In general, both STGNN models could profit the most when using CEs to characterize the spatial layout for functional interactions between brain regions. For the DCRNN, the test error was *MAE* = 0.1388 when incorporating no information from other brain regions in the network, and could be reduced to *MAE* = 0.1158 (for *K* = 1) when using CEs to model the information exchange within the brain network. To test whether incorporating information about structural connections significantly increases the prediction accuracy of our models, we at first recomputed the overall test MAE for each subject again. Then by using a paired *t* test, we find that, for both STGNN models (DCRNN and GWN) and both adjacency types (**A**_*SC*_ and **A**_*CE*_), the impact of structural modeling is positive (Cohen’s *d* > 1 for all comparisons) and significant (*p* ≤ 0.0001 for all comparisons), compared to the case in which it is not considered. Although the performance differences between the GWN and DCRNN are quite small in general, the DCRNN slightly outperformed with a test error of *MAE* = 0.1158 the GWN with a test error of *MAE* = 0.1211 at *K* = 1 (significant with *p* ≤ 0.0001, Cohen’s *d* = 0.49). In addition, the distribution of the test error across subjects and ROIs, with and without the structural modeling in STGNNs is illustrated in Supporting Information III in Figure S6. This demonstrates that around 17% more information on functional dynamics can be directly retrieved from nodes with similar context within the structural network. Using the SC to model transitions could only reduce the MAE of the DCRNN by 5% at *K* = 1. This observation supports the idea that structural node embeddings can strengthen the relationship between structural data derived from DTI with functional data observed in fMRI (Rosenthal et al., 2018). When applying a paired *t* test, the improvement of the prediction accuracy when using the CE similarity in comparison to the SC became for both, the DCRNN, and GWN model, significant with *p* ≤ 0.0001 at *K* = 1 (Cohen’s *d* = 1.45 for the DCRNN and *d* = 0.95 for the GWN). By inherently capturing higher order transitions in **A**_*CE*_, only a low walk order *K* is required to capture information from structurally connected ROIs. In this manner, this technique can help to efficiently reduce the number of necessary parameters to account for spatial dependencies in STGNN models.

### Model Accuracy and Network Scaling

In this section we study the prediction accuracy of the above introduced STGNN-based approaches and compare it to the VAR model, which is currently most often used for directed functional connectivity analysis (Barnett & Seth, 2013; Friston et al., 2013). In practicable applications, the amount of available fMRI data may vary depending on the project size and on the recruited subject cohort. Also, the size of the brain network of interest can range from a few specific areas in a single functional network to a large-scale whole-brain analysis. For this purpose we consider different scenarios in our following evaluations, by analyzing the model accuracies in dependence of the brain network size and the fMRI dataset size. We consider one larger subject dataset consisting of resting-state fMRI sessions from 50 different subjects, one medium sized dataset of 25 subjects, and one small dataset including data from 10 subjects. In addition, we vary the size of the analyzed brain network. The first network consists of 22 ROIs per hemisphere involved in visual processing as defined by the Glasser parcellation (Glasser et al., 2016) (a complete list of selected ROIs is provided in the Supporting Information II). The second network includes the regions within one hemisphere, and for that purpose the 180 regions within the right hemisphere included in the Glasser atlas were selected (Glasser et al., 2016). Finally, the whole-brain network of 360 regions in total was incorporated. As discussed in the Data Description section, windowed input and output time sequence pairs were created from the data, and the goal of the different models is accordingly to predict *T*_*f*_ = 60 TRs of neural activity from the past *T*_*p*_ = 60 activity values. We fitted the VAR model using the ordinary least squares method as implemented in the multivariate Granger causality toolbox (Barnett & Seth, 2013), and for each dataset we selected the VAR model with order *p* that achieved the best MAE on the test set, as outlined in more detail in the Vector Autoregressive Model section. The hyperparmeters used for the STGNNs are described in the methods part in the Model Training section. Further, for this comparison, the CE similarity **A**_*CE*_ with transition order of *K* = 1 was used to define the structural relations in the STGNN models, which has shown to improve the GNNs forecasting accuracy with low computational cost, as discussed in the section Comparison of GNN Architectures.

**Figure 4.**
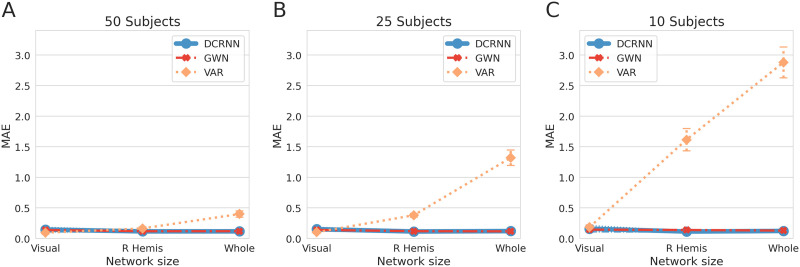
The figure shows a comparison of the model performances when varying the amount of data and the size of the network. The test MAE of the VAR is here depicted in orange, the MAE of the DCRNN in blue, and the error of the GWN in red. The overall error was computed as an average across brain regions, time steps and test samples. The error bars represent the standard deviation of the test error across subjects. (A) The test MAE using a dataset of 50 subjects is shown for the visual network, the network within the right hemisphere and the whole-brain network (Glasser et al., 2016). (B and C) The test performances in dependence of the network size using the 25 and 10 subject dataset, respectively.

Figure 4 shows the test accuracy of the VAR, DCRNN, and GWN model in dependence on the dataset size and brain network size. It can be observed in Figure 4A that if a large dataset of 50 subjects is available, all models are able to accurately predict the BOLD signal with a low test MAE, and a notable increase in the test error only appears for the VAR model, when it is fitted to the whole-brain network. Figure 4B shows the test MAE when data from 25 subjects is incorporated. In this case, the test error of the VAR model starts to increase noticeably when modeling activity distributions within one hemisphere and becomes quite large when including the whole-brain network. In contrast to these, the prediction accuracies of the DCRNN and GWN models remain stable in all cases. Finally, when only 10 subject datasets are available, the test MAE of VAR model is highly dependent on the analyzed network size, as illustrated in Figure 4C. The DCRNN and GWN models can still achieve a high accuracy, also when a limited amount of data are available and the network size is relatively large. In addition, this comparison of the models is replicated in Supporting Information IV using additional measures like *R*^2^ and the similarity of predicted FC states. After applying a paired *t* test, the differences between the DCRNN and GWN to the VAR were shown to be in all cases highly significant with *p* ≤ 0.0001 (Cohen’s *d* ≫ 1), except when the VAR is only fitted to the single visual network, where it still could make reliable forecasts.

**Figure 5.**
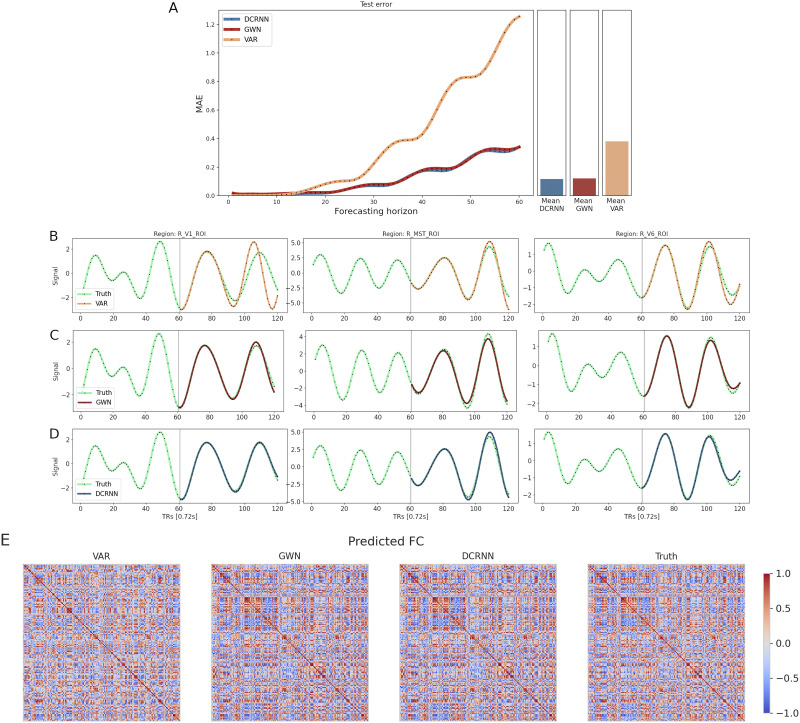
The prediction accuracy of the different models is presented in more detail for the 25 subject dataset and the brain network including the ROIs within the right hemisphere (Glasser et al., 2016). (A) The test MAE in dependence of the forecasting horizon is shown, computed as an average across test samples and brain regions. (B, C, and D) Examples of predictions generated by the VAR, GWN, and DCRNN model, respectively. The examples were chosen to be representative for the whole test set, by selecting only examples in which errors maximally deviate by 0.02 from the corresponding average test MAE of the models. (E) Examples of predicted FC states of the different forecasting models, including the true FC state on the right side. These representative examples deviated maximally by 0.005 from their average correlation to the true FC state.

To illustrate the prediction accuracies of the different models in more detail, an example of the predictions using the dataset including 25 subjects, and modeling the activity within one hemisphere, is shown in Figure 5. Figure 5A shows the MAE of the models computed as an average across test samples and ROIs in dependence of the forecasting horizon. In this case, within the first 15 predicted time steps all three models can generate very accurate predictions, but after that period the error of the VAR model starts to accumulate, while the GNN-based approaches remain considerably more stable and precise. The predicted BOLD signals of the different models in a few representative samples are shown in Figures 5B, C and D. Additionally, the predicted FC states **A**_*FC*_ ∈ ℝ^*N*×*N*^ were computed as the Pearson correlation between predicted BOLD signals of all *N* brain regions, and a comparison of representative predictions with the true FC state is illustrated in Figure 5E. The average correlation of the predicted FC state to the true FC state was for the VAR model *r*_*FC*_ = 0.635 on this dataset, while the GWN could achieve a correlation value of *r*_*FC*_ = 0.948, and the DCRNN a value of *r*_*FC*_ = 0.950. The overall FC similarity for all different datasets of all prediction models is given in Supporting Information IV Figure S8. Furthermore, in Supporting Information V in Figure S9 we performed the analysis on the same dataset using a more liberal frequency filtering within the 0.01–0.1 Hz frequency range. In this range, the signal dynamic becomes more complex, and we can observe an increase in the prediction error of the different models accordingly.

**Figure 6.**
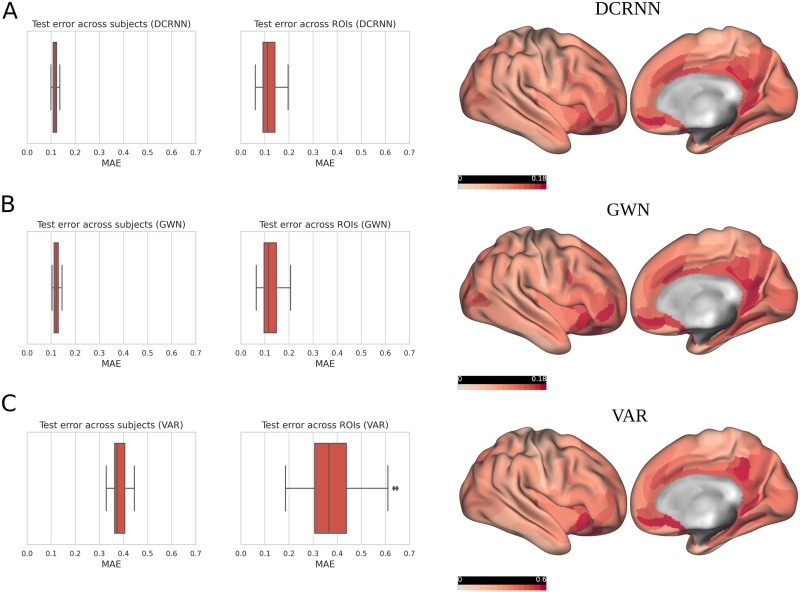
The distribution of the test error across subjects and brain regions is shown. (A) The MAE across subjects and brain regions of the DCRNN is first visualized in a boxplot on the left side. Additionally on the right side of the figure, the MAE values are projected onto the cortical surface within the right hemisphere, where the color map was linearly scaled between 0 and 0.18. (B) The distribution of the test MAE of the GWN is shown. (C) The MAE distribution of the VAR model. For the VAR, the color map was adjusted to account for larger error values by scaling it between 0 and 0.6.

In addition, we evaluated in more detail how the prediction errors are distributed across different subjects and different ROIs. Figure 6 shows the distribution of the test MAE of the DCRNN, GWN, and VAR model across subjects and in dependence of the brain region within the right hemisphere. For all three models we observe a consistently greater prediction error in the posterior cingulate cortex and medial orbitofrontal cortex, which could point toward a more complex BOLD dynamic in those regions. Alternatively, the prediction accuracy might also be affected by a lower signal-to-noise ratio observed in medial brain regions (Olman, Davachi, & Inati, 2009).

### Multimodal Directed Connectivity

In the Comparison of GNN Architectures section, different approaches have been investigated to model functional interactions between segregated regions in the brain network. The results showed that incorporating information on the spatial relation between ROIs in the form of the structural connectivity or connectome embedding similarity could considerably improve the prediction accuracy of the GNN models. This points out that the GNNs are able to learn relevant and functional informative transitions of neural activity on their structural spatial layout. Based on the idea of Granger causality (Granger, 1969) that the observation of one event *A* carries information about the occurrence of a future event *B*, this might represent initial evidence for a potentially causal relation between *A* and *B*. Due to the relatively low temporal sampling rate and physiological artifacts in fMRI (S. M. Smith et al., 2011; Webb et al., 2013), it still is a matter of discussion to what extent we can observe a *causal* relationship between brain regions in this imaging modality (Bielczyk et al., 2019; Mill et al., 2017; H. E. Wang et al., 2014). But the observation that activity in one region carries additional information among activity in another region in the brain can also go beyond simple undirected FC or SC, and we therefore refer to this kind of relation as *directed connectivity* in the following. Propagating the information between ROIs related to their SC or structural CE similarity has the potential to give us in this manner a multimodal perspective of such a directed relationship between different brain areas. For this purpose, we choose a perturbation base approach to reconstruct the amount of information individual ROI carries about other ROIs (Zeiler & Fergus, 2014). By learning a function *h*(·), the GNN models try to infer from an input sequence of neural activity states [**x**^(1)^, …, **x**^(*T*_*p*_)^] a sequence of future activity states [$\hat{x}$^(*T*_*p*_+1)^, …, $\hat{x}$^(*T*_*p*_+*T*_*f*_)^], whereby **x**^(*t*)^ ∈ ℝ^*N*^ denotes the activity at timestep *t* in all regions *n* = 1, …, *N*. To induce an artificial perturbation into the system of neural dynamics, we remove all activity in a certain ROI *n*′ by setting its activity values to the sample mean *x*_*n*′_ = 0. By using the perturbed time series as an input for our trained model *h*(·) the model generates then a prediction [$\hat{x}$′^(*T*_*p*_+1)^, …, $\hat{x}$′^(*T*_*p*_+*T*_*f*_)^]. To reconstruct the directed influence of ROI *n*′ on ROI *n*, we evaluate the overall difference between the original prediction and the prediction with perturbation in the input:

$$I_{n}\left(n^{\prime }\right)=\frac{1}{S}\sum_{s=0}^{S}\frac{1}{T_{f}}\sum_{t=0}^{T_{f}}\left|{\hat{x}}_{n}^{\left(t\right)}\left(s\right)-{{\hat{x}}^{\prime }}_{n}^{\left(t\right)}\left(s\right)\right|$$ (2)

where *I*_*n*_(*n*′) denotes the impact of ROI *n*′ on *n*. Further, ${\hat{x}}_{n}^{\left(t\right)}$(*s*) and ${{\hat{x}}^{\prime }}_{n}^{\left(t\right)}$(*s*) denote the predictions in ROI *n* with and without the perturbation in *n*′ of one test sample *s* at time step *t*. Note that this artificial perturbation approach has only the goal of making spatial relations between ROIs in STGNN models explainable, and should not be equated with the effect of an experimental perturbation applied to the human brain, like, for example, induced by transcranial magnetic stimulation.

**Figure 7.**
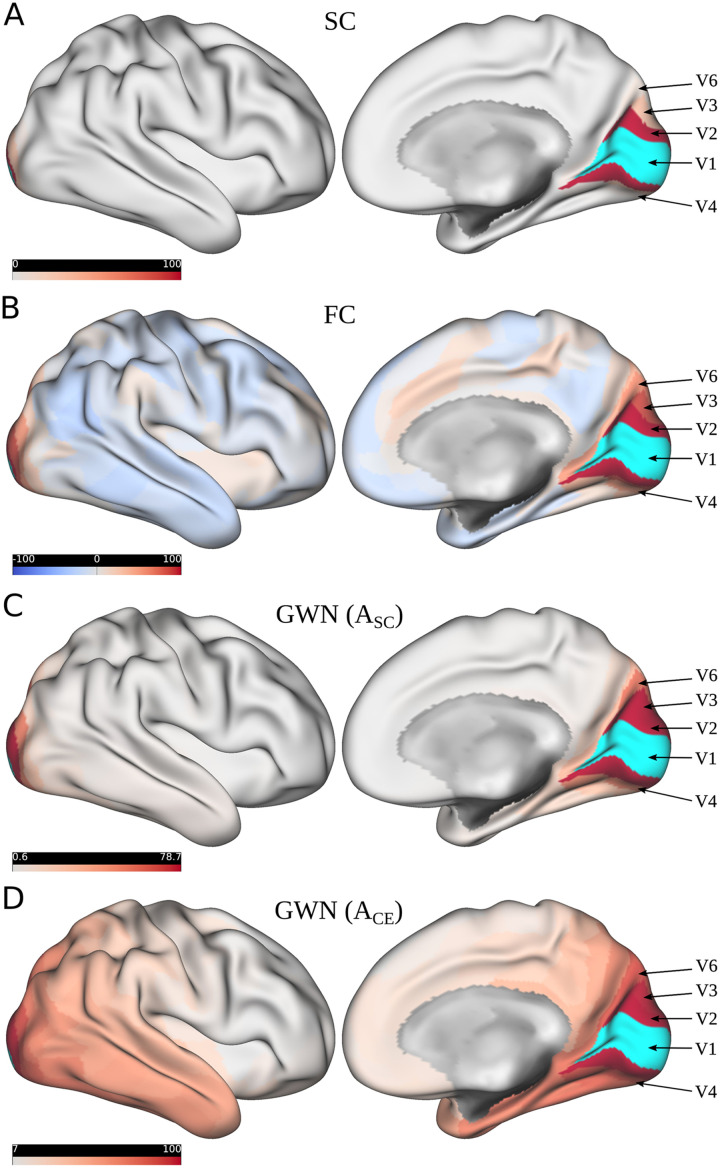
Different types of connectivity are illustrated between V1 and all other regions within the right brain hemisphere. (A) The structural connectivity is shown, whereby the target region V1 is marked in light blue and the connectivity strength is encoded in red. (B) The correlation-based functional connectivity is illustrated, which was computed as an average across subjects. (C) The measures of influence **I**(*n*′), derived from the GWN model using the SC for information propagation. (D) The influence when incorporating CEs for the information exchange between ROIs. The values of the connectivity measure were linearly mapped between 0 and 100 (and between −100 and 100 for FC). The default scaling of the color values provided by the *connectome workbench* (version 1.4.2) was used, adjusting the color map between the 2*th* and 98*th* percentile of the values respectively.

In the following we compare this proposed measure of directed influence **I**(*n*′) to the classical undirected types of brain connectivity. First, we compare it to structural connectivity as derived from DTI, characterized by the number of fiber tracks connecting two brain regions. Then, we incorporate functional connectivity, defined as the Pearson correlation of functional activity time courses between two areas. We employ the above introduced GWN model to obtain a multimodal measure of directed connectivity **I**(*n*′), first using the SC as substrate for information propagation, captured in **A**_*SC*_, and then also employing the similarity of CEs, represented by **A**_*CE*_. In the following example, we study the connectivity of V1 within the right hemisphere by incorporating data of 25 subjects. For this purpose, a perturbation was induced into the target region V1, and the impact of this perturbation on all other 179 regions within the right hemisphere was then characterized by computing the measure of directed influence **I**(*n*′), as defined in Equation 2. These values for directed connectivity strength can then be visualized by projecting them onto the 179 other areas of the cortical surface. For the following comparison, all connectivity values were rescaled by normalizing them between 0 and 100. At first, in Figure 7A the structural connectivity between V1 and all other 179 regions within the right hemisphere is depicted. The target region V1 is marked here in light blue, and the strength of connectivity to all other regions is encoded in red. Figure 7A shows that we can mainly observe a pronounced structural connectivity between V1 and V2 and some structural connections leading to V3. Figure 7B shows the undirected functional connectivity in resting state. In this type of connectivity, we can observe predominantly correlations to the functional activity in V2 and V3, but also a considerable connection strength to V3, V4, and V6. In Figure 7C the directed connectivity strength **I**(*n*′) is depicted, when using the SC as spatial backbone for the information exchange between brain regions in the GNN. In comparison to the SC, in this variant of brain connectivity we can observe in addition to V2 also a more pronounced relationship to areas V3 and V4, and to some anatomically more distant areas like V6 and the ventromedial visual area VMV1. This multimodal type of connectivity also reflects the role of indirect structural connections by modeling higher order transitions on the structural scaffold captured by the STGNN model. As an alternative to the SC, in Figure 7D the directed connectivity patterns when using CE similarity as the spatial layout in the GWN are displayed. Here we can see an even stronger integrity of V1 within the visual network, which is in agreement with the observation that CEs capture higher order topological information of anatomical connectivity (Rosenthal et al., 2018). Figure S10 in Supporting Information VI shows additionally the spatial relations learned by the DCRNN model. Here we can observe a pronounced similarity to the directed connectivity pattern learned by the GWN architecture, showing additionally strong relations to areas like V3 and V4. Based on this observation, such a GNN-based connectivity approach can serve as a link between structural and functional connectivity, and as such they can provide a multimodal perspective on directed influences between individual areas in brain networks. So far, we have only studied the effect of a single artificial perturbation in V1 directed to all other regions within the right hemisphere. This approach can further be extended to sample a full connectivity network, by systematically inducing perturbations into all regions of interest for the analysis, and then systematically observing the effect on the other network regions. In Figure S11 in Supporting Information VI, we studied the effects of perturbations induced in some different additional areas of the visual network based on this approach. In this manner, this technique can allow us to reconstruct the directed spatial relations between brain areas captured in STGNN models, and could be applied in practical applications by, for example, comparing these connectivity patterns between different conditions in task-based fMRI, or studying the difference between healthy and diseased brain states.

A study of Shinn et al. (2021) demonstrated that FC topology in resting-state fMRI is shaped by and can be predicted from spatial autocorrelations and temporal autocorrelations, as typically observed in fMRI data. To also investigate to what extend STGNN-based connectivity patterns are related to such correlations, we computed the temporal autocorrelation as the Pearson correlation between the BOLD signal values and its lagged values with different lag orders *j*, depicted in Figure S12 in Supporting Information VI. We could observe a relatively high temporal correlation of 0.89 around lag order *j* = 14, and of 0.62 around lag order *j* = 27, which shows that within these first 30 TRs of the signal, such temporal autocorrelations can still be detected. In section Model Accuracy and Network Scaling we could show that STGNNs were also able to make reliable long-term predictions of the BOLD signal up to a horizon of 60 TRs, which demonstrates that STGNNs capture properties of neural activity dynamics that go clearly beyond the range that is shaped by temporal autocorrelations. In addition, spatial autocorrelations can also play a distinctive role in shaping FC network properties (Shinn et al., 2021). The analysis in section Comparison of GNN Architectures showed that by modeling the information exchange between structurally connected brain regions in the spatial domain, the prediction accuracy of the STGNNs could be significantly improved, in comparison to the null models, which incorporated no spatial dynamics. The observation that spatially connected regions contain some *additional* relevant information on functional dynamics points out that spatial interactions captured in STGNNs go beyond simple correlation-based spatial network relations. A more detailed comparison of the individual differences and similarities between correlation-based FC and the STGNN-based connectivity pattern is additionally provided in a bar plot in Supporting Information VI (Figure S13).

## CONCLUSION

In this study we have compared different STGNN architectures for learning the spatiotemporal dynamics in brain networks. First, in the section Comparison of GNN Architectures we studied different mechanisms for learning the temporal dynamics in the BOLD signal. We could show that an RNN-based model and a WN-based model exhibit very similar capabilities in learning the temporal characteristics in neural activity time series. Despite their conceptual differences in their architectures, they demonstrated almost the exact same prediction accuracy, which indicates that they are both very consistent in capturing the temporal information in the data. As an alternative, we also studied TAtt mechanisms to learn temporal characteristics of neural signals. The TAtt model showed to be less suitable to model the dynamics in the BOLD signal with a limited amount of fMRI data. Despite incorporating techniques into the TAtt model that in general stabilize the learning, like residual connections and batch normalization (He, Zhang, Ren, & Sun, 2016; Ioffe & Szegedy, 2015), its prediction error was considerably higher in comparison to the RNN- and WN-based approach. This indicates that the geometric assumptions that are realized by the temporally structured inference in the RNNs and WNs based on either recurrent computations or causal convolutions can contribute to the learning of the temporal characteristics of the BOLD signal. We then studied the impact of adding spatial dependencies to the temporal models, realized by invoking graph convolution operations. We have compared different spatial layouts for information propagation between ROIs, and therefore included either the structural connectivity (**A**_*SC*_), the CE similarity (**A**_*CE*_), or a self-adaptive adjacency matrix (**A**_*Adap*_) into the STGNN models. While the model performance of the GWN and DCRNN steadily improved with higher walk orders *K* on the anatomical substrate, we could observe a more pronounced improvement already when using CEs with a walk order of only *K* = 1. This embedding strategy turns out to be therefore also interesting in applications of STGNNs, because it helps to effectively incorporate indirect structural connections with low computational cost. In addition, the observed characteristics of CEs in our application support the ideas of Rosenthal et al. (2018), which showed in their study that embeddings of the structural network can naturally capture higher order topological relations between ROIs within the structural layout. In our context of modeling spatiotemporal dynamics, this method also proved to strengthen the relationship between brain structure and functional dynamics. Learning all *N*^2^ connections of the underlying structural graph during the model training has been shown to be challenging for the STGNN models, in case no prior knowledge is provided to them in the form of the anatomical brain connectivity. While such highly parameterized artificial neural network models can be in theory quite flexible in learning complex relations (Brüel Gabrielsson, 2020; Hornik, Stinchcombe, & White, 1989), often the decisive limitation is the successful optimization of the parameters during model training (Dauphin et al., 2014). In the discussed applications of STGNNs in fMRI, where the amount of training data is most often quite limited, prior knowledge in the form of the anatomical graph structure has been shown to considerably support the learning of spatial relations between brain areas captured in STGNN models.

So far, methods based on biophysical modeling (Deco et al., 2012, 2013; Honey et al., 2009; Messé et al., 2014, 2015; Messé, Rudrauf, Giron, & Marrelec, 2015), graph theory (Abdelnour et al., 2018; Becker et al., 2018; H. Liang & Wang, 2017; Lim et al., 2019), or machine learning (Amico & Goñi, 2018; Deligianni et al., 2016; Rosenthal et al., 2018; Sarwar et al., 2021) have contributed already numerous valuable insights into the structure-function relation in brain networks, and could highlight the role and importance of the structural connectome in shaping functional connectivity patterns. While the majority of approaches studying the structure-function relationship infer brain dynamics by fitting the models to empirically observed FC patterns, STGNNs provide us with a possibility to directly predict the observed neural activity states. Similar to some other recently proposed predictive models (Singh, Braver, Cole, & Ching, 2020; Suárez et al., 2021), this principle can allow us to investigate additional interesting aspects of dynamic brain functions. As discussed in section Comparison of GNN Architectures, this could enable us, for example, to study directly the amount of information on the activity of one ROI that is contained in the activity of other structurally connected ROIs. For a comparison with other currently used approaches investigating SC-FC mappings, the predicted BOLD signal states of STGNN can be used then again to reconstruct predictions for FC states, as shown in Figure 5E. The relatively high accuracy in predicting empirical FC states already points out the potential of STGNNs in this field. Moreover, in comparison to other currently popular approaches used in this area (Messé et al., 2015), by learning localized graph filters in STGNNs, their forecasting accuracy is also robust with regards to the brain network size. While such highly parameterized artificial neural network models appear to be promising for achieving high prediction accuracies of FC states (Sarwar et al., 2021), they cannot provide us with the same mechanistic insights into physiological processes as biophysically inspired models. Still, they can be used to supplement current biophysically inspired models, for studying different aspects of the structure-function relationship from a novel data-driven perspective. A more comprehensive comparison of these different new approaches, evaluating in detail their interrelations like in the study of Messé et al. (2015), could be thereby interesting for future studies in this area.

In the Model Accuracy and Network Scaling section, we have compared the STGNN models to a VAR model, which is currently most often used in Granger causality analysis for inference of directed relationships between brain regions (Barnett & Seth, 2013). We evaluated the accuracy of the different approaches on a variety of brain network sizes and dataset sizes to account for different possible scenarios in their application in fMRI studies. The results showed that if a sufficiently large cohort of 50 subjects is available, also a VAR model is able to make very reliable long-term predictions, and only for a large network consisting of *N* = 360 there is a notable increase in the prediction error. But the dependency of the accuracy on the network size *N* becomes more apparent when data from only 25 subjects are used to fit the VAR model, and when only 10 subjects are available, the error grows strongly with *N*. This demonstrates that a VAR is a very reliable and fast model for fMRI studies with a sufficiently large test subject size and for connectivity studies including a limited amount of predefined regions. However, it can be desirable in some cases to include a larger amount of brain areas into the connectivity analysis, in order to avoid omitting relevant areas in the network of interest. Also, in MRI studies it can be very costly and time-consuming to collect a large amount of data, which is, for example, especially challenging in studies on rare neurological disorders. A classical VAR model fits a parameter for every possible connection between the *N* regions in a network, so that the number of parameters in a VAR-based approach grow strongly with an order of *N*^2^. In contrast thereto, STGNNs utilize prior information in the form of the anatomical connectivity, and then model the functional information exchange based on this underlying structural substrate. By incorporating graph convolution operations in STGNNs, the amount of parameters only linearly scale with walk order *K*, which can even be chosen to be *K* = 1, if higher order structural relations are already expressed in an adjacency matrix derived from connectome embeddings (**A**_*CE*_). This property allowed STGNNs to make very robust inferences also on large networks and when only limited data are available, thereby providing a flexible method for various connectivity analysis scenarios.

Finally in the Multimodal Directed Connectivity section, we studied the individual spatial interactions within the brain network that were learned by the STGNN models. By integrating information on the anatomical connectivity into the GNN-based models, we could derive a multimodal connectivity measure for directed relationships between brain regions. When comparing this measure of influence to the original structural connectivity, we can observe that STGNNs have learned to include transitions along higher order structural connections in the network. The models could infer links between *V*1 and *V*2, but additionally strong connections to *V*3 and *V*4. Especially when incorporating the CE-based similarity **A**_*CE*_ to define spatial node relations in the STGNN models, we can observe a high integration of *V*1 within the visual system. However, due to the relatively low temporal sampling rate in fMRI (Friston et al., 2013), and the indirect measurement of neural signals based on their hemodynamic response (Webb et al., 2013), one should also be aware of these limitations in the inference of directed and potentially causal connections in fMRI studies (S. M. Smith et al., 2011). Our lag-based predictive approach based on STGNN models might therefore also be affected by the same limitations as classical Granger causality in fMRI. On the other hand, a combined fMRI-MEG study by Mill et al. (2017) and different computational simulations of fMRI data (Duggento et al., 2018; Seth et al., 2012; H. E. Wang et al., 2014; Wen, Rangarajan, & Ding, 2013) could establish evidence that Granger causality is still able to identify meaningful directed relationships between brain areas in fMRI, despite the indirect measurements based on the hemodynamic response. As an alternative, deconvolution-based approaches can have the potential to infer from the measured BOLD signals the underlying neural time series (Bush et al., 2015; Mill et al., 2017) for assessing *effective* brain connectivity, rather than only estimating *directed* functional connectivity. But the estimation of the underlying hemodynamic response from the data might come with the cost of introducing additional assumptions and uncertainties (Bielczyk et al., 2019; Roebroeck, Formisano, & Goebel, 2011). A more detailed discussion on considerations concerning Granger causality, and, in general, causal inference in fMRI, is provided in the comprehensive review of Bielczyk et al. (2019), as well as in the perspective on FC and its variants by Reid et al. (2019). Despite these current limitations in fMRI, a multimodal GNN-based approach can allow us to join structural and functional imaging data in a new manner, and reveals thereby potential for supplementing current analysis methods in brain connectivity research by studying such directed relations under a novel perspective (Reid et al., 2019).

In conclusion, in our study we found that the DCRNN and GWN architecture are both suitable for the task of functional dynamics inference. Using CEs to characterize the structural similarities between brain regions could further improve their prediction accuracy. Their robust scaling properties and the possibility to combine the information in structural and functional MRI data reveal the potential of STGNNs in the field of brain connectivity analysis. Besides their applications in fMRI, other functional neuroimaging techniques like electroencephalography (EEG) or magnetoencephalography (MEG) might be interesting for analyzing temporal dynamics with STGNNs in the high-frequency range. While in this presented approach we only incorporated a single temporal feature (the BOLD signal) into the STGNNs, in general, such a flexible data-driven approach could be expanded to account for different types of data and annotations. For example, the activity measured in a combined EEG-fMRI experiment (Abreu, Leal, & Figueiredo, 2018; Mele et al., 2019) could be also simultaneously integrated in STGNNs as different temporal features, or adding the temporal response of a subject could be helpful to better predict activity patterns in task-based fMRI. Also, alternative structural imaging techniques like neurite orientation dispersion and density imaging (NODDI) (Zhang, Schneider, Wheeler-Kingshott, & Alexander, 2012) might capture additional aspects of the brain structure, which could be included as structural information in STGNN-based models. In clinical applications multimodal STGNNs could be interesting for studying how the relationship between structure and function is affected in the diseased brain (Panda et al., 2021), or which impact a structural lesion might have on the functional organization of the brain network (Alstott, Breakspear, Hagmann, Cammoun, & Sporns, 2009). Still, research on GNNs is a relatively new field in machine learning, and recent developments in this field can make interesting contributions to our understanding of information processing in brain networks (de Haan, Cohen, & Welling, 2020; Schnake et al., in press).

## MATERIALS AND METHODS

### Dataset

The MRI dataset used in our study is provided by the HCP data repository (Hodge et al., 2015; Van Essen et al., 2013). As part of the HCP protocol, the study participants gave written informed consent to the HCP consortium. The MRI scanning protocols were approved by the Institutional Review Board at Washington University in St. Louis. We incorporated data of the *S1200 release*, which provides data from resting-state fMRI sessions, each with a duration of 14.4 minutes, whereby 1,200 volumes were sampled per session. The data was acquired with customized Siemens Connectome Skyra magnetic resonance imaging scanners with a field strength of *B*_0_ = 3*T*, using multiband (factor 8) acceleration (Feinberg et al., 2010; Moeller et al., 2010; Setsompop et al., 2012; J. Xu et al., 2012). A gradient-echo echo-planar imaging (EPI) sequence with a repetition time *TR* = 720 ms and an echo time *TE* = 31.1 ms was used. The field of view of the fMRI sequence was FOV = 208 mm × 180 mm and in total *N*_*s*_ = 72 slices with a slice thickness of *d*_*s*_ = 2 mm were collected, containing voxels with an isotropic size of 2 mm. The preprocessing of the HCP fMRI data includes corrections of gradient-nonlinearity-induced distortions, registration to a single-band reference image to account for subject motion and registration to the structural T1w image (Fischl, 2012; Glasser et al., 2013; Jenkinson, Bannister, Brady, & Smith, 2002; Jenkinson, Beckmann, Behrens, Woolrich, & Smith, 2012). Further, ICA-FIX was applied to automatically classify and remove artifactual components in the resting-state fMRI data (Griffanti et al., 2014; Salimi-Khorshidi et al., 2014; S. M. Smith et al., 2013). Finally, the volumetric fMRI images are mapped into the CIFTI grayordinate space and Gaussian surface smoothing with a FWHM of 2 mm is performed. A detailed description of the standard minimal preprocessing pipelines of the HCP can be found in Glasser et al. (2013). In a next step to define our brain network, the multimodal parcellation proposed by Glasser et al. (2016) was applied, which divides the cortical surface into 180 segregated areas per hemisphere. The BOLD signal within each area was averaged, to obtain the temporal activity evolution for each node in our brain network. For this study, we considered it useful to apply global signal regression in our preprocessing (Power, Plitt, Laumann, & Martin, 2017). Firstly, in a systematic comparison of different preprocessing methods to address motion artifacts Ciric et al. (2017) could show that an ICA-based denosing in combination with global signal regression is among the most effective methods to reduce movement artifacts. This result is in line with the study of Burgess et al. (2016), investigating the effect of ICA-FIX in combination with global/grayordinate signal regression on resting-state fMRI data provided by the HCP. Furthermore, in our study of functional interactions between specific brain regions, the objective was to extract the additional information, which certain regions contain about the activity in other regions. Therefore, local interactions rather than global modulations in the signal were the main interest for our analysis (Power et al., 2017). The time courses were then band pass filtered in the 0.04–0.07 Hz frequency range. In a summary of several different studies that account for different artifacts in the BOLD signal related to MRI scanner drift in the frequency range below 0.015 Hz (A. M. Smith et al., 1999), respiratory and cardiac frequencies around 0.3 Hz and 1–2 Hz respectively (B. Biswal, DeYoe, & Hyde, 1996), and fluctuations in arterial carbon dioxide level around 0.0–0.05 Hz (Wise, Ide, Poulin, & Tracey, 2004), the study of Glerean et al. (2012) identified the 0.04–0.07 Hz frequency band to be most reliable and relevant for gray matter activity in resting-state fMRI (Achard et al., 2006; Buckner et al., 2009; Zou et al., 2008). To additionally ensure that the low-frequency signals are not mainly related to respiratory artifacts, we studied the respiratory signals recorded with a Siemens respiratory belt during resting-state fMRI, as provided by the HCP (S. M. Smith et al., 2013). The average respiratory frequency spectrum is depicted in Supporting Information VII in Figure S14, and we can observe that respiratory fluctuations are mainly present in the higher frequency range around 0.28 Hz in this resting-state fMRI dataset. In addition, the different models were tested on data incorporating a more liberal frequency filtering within the 0.01–0.1 Hz range, as presented in Supporting Information V.

In the S1200 release, diffusion MRI data was collected in six runs, whereby approximately 90 directions were sampled during each run, using three shells of *b* = 1,000, 2,000, and 3,000 s/mm^2^, with additionally 6 *b* = 0 images (Sotiropoulos, Jbabdi, et al., 2013). A spin-echo EPI sequence was incorporated with repetition time *TR* = 5,520 ms, echo time *TE* = 89.5 ms, using a multiband factor of 3. In total *N*_*s*_ = 111 slices were collected, with field of view FOV = 210 mm × 180 mm and an isotropic voxel size of 1.25 mm. The minimal preprocessing pipeline of the HCP includes intensity normalization across runs, EPI distortion correction using the FSL5 “topup” tool, correction of eddy current–induced field inhomogeneities and head motion artifacts using the FSL5 “eddy” tool, and finally includes gradient nonlinearity corrections and registration to the structural T1w image (Andersson, Skare, & Ashburner, 2003; Andersson & Sotiropoulos, 2015a, 2015b; Glasser et al., 2013; Sotiropoulos, Moeller, et al., 2013). More details on the minimal preprocessing of the HCP diffusion MRI are described in Glasser et al. (2013). To reconstruct the anatomical connection strengths between regions within the multimodal parecellation (Glasser et al., 2016), the MRtrix3 software package was incorporated (Tournier et al., 2019). Multishell multitissue-constrained spherical deconvolution (Jeurissen et al., 2014) was applied to obtain response functions for fiber orientation distribution estimation (Tournier, Calamante, & Connelly, 2007; Tournier, Calamante, Gadian, & Connelly, 2004). Then 10 million streamlines were created using anatomical constrained tractography (R. Smith, Tournier, Calamante, & Connelly, 2012). Finally, spherical deconvolution–informed filtering was used (R. Smith, Tournier, Calamante, & Connelly, 2013), reducing the number of streamlines to 1 million. The strength of SC was defined as the number of streamlines connecting two brain regions, normalized by the region volumes. The group structural connectivity matrix **A**_*SC*_ was obtained as the average SC across the first 10 subjects, because the variance in the SC strength was relatively low across subjects (Zimmermann, Griffiths, Schirner, Ritter, & McIntosh, 2018), while probabilistic tractography methods are computationally demanding. For the HCP dataset, including only young healthy subjects, the similarity of the SC across subjects was quite high, and the Pearson correlation coefficient between SC values of the 10 subjects was on average 0.91. But when comparing very different subject cohorts, like healthy and diseased subjects, the anatomical connectivity can differ considerably between those cohorts, and the SC matrix should then be computed for every studied group individually.

### Graph Neural Networks

Different brain areas communicate via bioelectrical signals transmitted along neuronal axons and collected by neuronal dendrites. Spatiotemporal GNNs provide a novel possibility to incorporate such a structural scaffold into a graph-based prediction model (Wein, Malloni, et al., 2021). Due to cognitive information processing in the brain, the spatial interactions of the activity distribution changes dynamically. Spatiotemporal GNNs thus encompasses both the information about the layout of the physical scaffold encoded by the graph structure and the dynamical information about temporal activity correlations. Recently, we used a DCRNN architecture to model the spatiotemporal brain dynamics in resting-state fMRI (Wein, Malloni, et al., 2021). In this study, spatial dependencies of brain activities were modeled via diffusion convolution operations based on the anatomical connectivity and the temporal dynamics of the graph signal were captured in an RNN-based model architecture (Y. Li et al., 2018). In our current study, we evaluate some alternative spatial and temporal approaches to model dynamics in brain networks. In addition to RNNs, a CNN-based architecture for temporal modeling has been introduced by Wu et al. (2019). These authors built upon WaveNets (van den Oord et al., 2016) and stack dilated causal convolution layers to capture long-range temporal dependencies. Dilated convolutions support exponentially growing receptive fields in deeper layers of the network and allow us to handle long-range temporal sequences efficiently (van den Oord et al., 2016). In addition to the temporal processing based on RNNs and CNNs, we also follow ideas expressed in attention networks and incorporated a relevance score that was computed in temporal attention layers (Vaswani et al., 2017; Zheng et al., 2020).

Based on these temporal approaches, we further study different concepts for representing the spatial dependency between brain regions. First, we integrated the SC reconstructed from DTI to represent the anatomical substrate for information propagation in graph convolution operations. Then, we additionally incorporated CEs of the structural graph to inherently capture higher order relations between ROIs. Finally, we used no predefined spatial layout and treated the spatial connection strengths between ROIs as free parameters. These different spatiotemporal GNN architectures have to the best of our knowledge not been applied yet to analyze the dynamics of brain networks, and in our study we investigate their effectiveness in spatiotemporal modeling of functional MRI.

### Preliminaries

Let us represent the brain network as a graph. Every specific brain area or region of interest (ROI), then forms a node in the graph. Let these *N* ROIs form a graph 𝒢 = (𝒱, 𝓔, **A**_*w*_) encompassing *N* vertices, that is, the meta-voxels or ROIs, and a set 𝓔 of edges connecting the vertices *v*_*n*_, *v*_*n*′_. The graph structure can then be captured in a weighted adjacency matrix **A**_*w*_ ∈ ℝ^*N*×*N*^, whose entries *w*_*nn*′_ provide the connection strengths between vertices *v*_*n*_ and *v*_*n*′_ and implicitly define the spatial structure of the graph. As introduced above, in our study we compared three different variants to define the spatial relationship between ROIs. Once we incorporated the SC derived from DTI data as an adjacency matrix **A**_*SC*_, we next employed CE to additionally capture higher order topological features in SC represented by **A**_*CE*_, and finally we treated the spatial relations as adaptive learnable parameters **A**_*Adap*_ in the GNN models. The dynamics of the graph signal is then represented by the time-varying neural activity obtained from functional imaging data. Let us first assume that each node of the graph is associated with a single feature represented by the BOLD activity. By considering voxel time series of brain activity maps, then all data can be collected into a data matrix **X** = (**x**^(1)^, …, **x**^(*T*)^) ∈ ℝ^*N*×*T*^ with **x**^(*t*)^ ∈ ℝ^*N*^. Given *N* ROIs, taken from a brain atlas and each represented by a meta-voxel, and considering *T* time points for each meta-voxel time series, which represents the activation time course of one of the ROIs, then we have, for the BOLD feature represented at node *n* a related graph signal matrix or BOLD feature matrix:

$${𝒳}_{::m}\equiv X^{\left(m\right)}=\left(x_{1}^{\left(m\right)}\ldots x_{T}^{\left(m\right)}\right)=\left(\begin{array}{ccc} x_{11}^{\left(m\right)} & \cdots & x_{1T}^{\left(m\right)}\\ \vdots & x_{nt}^{\left(m\right)} & \vdots \\ x_{N1}^{\left(m\right)} & \cdots & x_{NT}^{\left(m\right)} \end{array}\right)\in {\mathbb{R}}^{N\times T}$$ (3)

Note that the columns $x_{t}^{\left(m\right)}$ ∈ ℝ^*N*^ of the data matrix describe the activation of all ROIs at any given time point 1 ≤ *t* ≤ *T*, while its rows ${\tilde{x}}_{n}^{\left(m\right)}$ represent the meta-voxel time course of every single ROI 1 ≤ *n* ≤ *N*. More generally, if nodes not only represent a single feature *m*, like the input BOLD signal, but an *M*-dim feature vector 𝒳_*nt*:_ ∈ ℝ^*M*^, then we obtain a feature tensor 𝒳 ∈ ℝ^*N*×*T*×*M*^, whose frontal, lateral (vertical), and horizontal slices, respectively, read 𝒳_::*m*_ ∈ ℝ^*N*×*T*^, 𝒳_:*t*:_ ∈ ℝ^*N*×*M*^, and 𝒳_*n*::_ ∈ ℝ^*T*×*M*^.

In addition to above frontal slices 𝒳_::*m*_ ≡ **X**^(*m*)^ of the data tensor 𝒳, we thus have the lateral tensor slices:

$${𝒳}_{:t:}\equiv X^{\left(t\right)}=\left(x_{1}^{\left(t\right)}\ldots x_{M}^{\left(t\right)}\right)=\left(\begin{array}{ccc} x_{11}^{\left(t\right)} & \cdots & x_{1M}^{\left(t\right)}\\ \vdots & x_{nm}^{\left(t\right)} & \vdots \\ x_{N1}^{\left(t\right)} & \cdots & x_{NM}^{\left(t\right)} \end{array}\right)\in {\mathbb{R}}^{N\times M}$$ (4)

and the horizontal tensor slices:

$${𝒳}_{n::}\equiv X^{\left(n\right)}=\left(x_{1}^{\left(n\right)}\ldots x_{M}^{\left(n\right)}\right)=\left(\begin{array}{ccc} x_{11}^{\left(n\right)} & \cdots & x_{1M}^{\left(n\right)}\\ \vdots & x_{mt}^{\left(n\right)} & \vdots \\ x_{T1}^{\left(n\right)} & \cdots & x_{TM}^{\left(n\right)} \end{array}\right)\in {\mathbb{R}}^{T\times M}$$ (5)

Note that the column fibers of the data tensor 𝒳_:*tm*_, denoted as $x_{t}^{\left(m\right)}$ represent, at every time point *t*, the distribution of the activity of feature *m* across all nodes *n* of the graph. Correspondingly, the row fibers of the tensor 𝒳_*n*:*m*_, denoted as $x_{n}^{\left(m\right)}$, represent the time course of every feature *m* at node *n*. Finally, the tube fibers of the tensor 𝒳_*nt*:_, denoted as $x_{t}^{\left(n\right)}$, represent the distributions of features at every node *n* and time point *t*. This notation will in the following provide the framework to introduce the different techniques to model either dependencies between nodes *n*, time *t*, or features *m*.

### Spatial Dependencies

#### Diffusion convolution.

In the following we provide a short introduction on a variant of graph convolution denoted as *diffusion convolution* in the context STGNNs (Y. Li et al., 2018; Wu et al., 2021). The information flow in the underlying graph 𝒢 = (𝒱, 𝓔, **A**_*w*_) is considered as a stochastic random walk process modeled by a state transition matrix **T** = **D**^−1^**A**_*w*_ = (**ŵ**_1_ … **ŵ**_*N*_) where **A**_*w*_ represents a weighted adjacency matrix. The diagonal node degree matrix is given by:

$$D=\text{diag}\left(A_{w}1\right)$$ (6)

where **ŵ**_*n*_ = (*ŵ*_1*n*_ … *ŵ*_*Nn*_)^*T*^ ∈ ℝ^*N*^ ∀ *n* = 1, …, *N* with *ŵ*_*nn*′_ = *w*_*nn*′_/∑_*n*′_ *w*_*nn*′_ denoted normalized edge strengths. State transitions were modeled as a diffusion process on an unstructured graph. The former was represented by a random walk Laplacian:

$$L_{rw}=I-T=U\hat{\Lambda }U^{T}=U\left(I-\Lambda \right)U^{T}=I-{U\Lambda U}^{T}$$ (7)

where the transition operator **T** was replaced by its eigen-decomposition with **U** the matrix of eigenvectors and **Λ** the diagonal matrix of eigenvalues. Hence, the set of eigenvectors provided an orthogonal basis system for the spatial representation of the brain graph. With the help of these eigenvectors **u**_*n*_ the spatial structure of the graph could be implemented. A spectral representation in combination with the convolution theorem then provided a definition of the graph convolution operator 𝒢_*C*_ (Shuman, Narang, Frossard, Ortega, & Vandergheynst, 2013), which served to compute the spatial convolution of the input signal and a spatial filter kernel to yield the output of the *ℓ*-th convolution layer as:

$$\begin{array}{l} y_{t}^{\left(q\right)}={U\Theta }_{\omega }^{\left(q\right)}x_{\omega }^{\left(m\right)}={U\Theta }_{\omega }^{\left(q\right)}U^{T}x_{t}^{\left(m\right)}\\ \;\approx \sum_{k=0}^{K}\theta_{k}^{\left(q\right)}\left(\omega \right)T^{k}x_{t}^{\left(m\right)} \end{array}$$ (8)

Here the approximation resulted from a power series expansion of the convolution kernel with respect to the eigenvalue matrix **Λ** of the transition operator **T** (Defferrard et al., 2016; Wein, Malloni, et al., 2021). Finally, considering a CNN architecture and applying the graph convolution operator 𝒢_*C*_, the filtered input signal $y_{t}^{\left(q\right)}$ was transformed with an activation function *σ*(·) to yield the output $h_{t}^{\left(q\right)}$ of each of the *q* ∈ {1, …, *Q*} graph convolution layers as follows:

$$h_{t}^{\left(q\right)}=\sigma \left(y_{t}^{\left(q\right)}\right)=\sigma \left(\sum_{k=0}^{K}\theta_{k}^{\left(q\right)}\left(\omega \right)T^{k}x_{t}^{\left(m\right)}\right)$$ (9)

Hereby $x_{t}^{\left(m\right)}$ ∈ ℝ^*N*^ denotes the *m*-th input feature component at time *t*, $h_{t}^{\left(q\right)}$ ∈ ℝ^*N*^ the corresponding output component of the *q*-th convolution channel, $\Theta_{k}^{\left(q\right)}$ ∈ ℝ^*N*^ parameterizes the *q*-th convolutional kernel of order *k* and *σ*(·) denotes any suitable activation function. Note that for deeper convolution layers *ℓ* > 1, *ℓ* = 1, …, *L*, the input to the convolution *ℓ* layer is given by the output component of the convolution layer *ℓ* − 1 instead of the input signal. In summary, these graph convolution layers can learn to represent graph-structured data and could be trained with gradient descent–based optimization techniques.

#### Structural connectivity.

One possibility to define the spatial layout of the brain network characterized by the weighted adjacency matrix **A**_*w*_ is to directly incorporate the structural connection strength as reconstructed from DTI data. The weights *w*_*nn*′_ in our adjacency matrix would accordingly reflect the number of fibers connecting two brain regions *n* and *n*′, derived from probabilistic fiber tracking (Tournier et al., 2004). This type of structural adjacency relation is denoted as **A**_*SC*_ ∈ ℝ^*N*×*N*^. The acquisition parameters of the DTI data and the structural connectome generation are outlined in detail in the Dataset section.

#### Connectome embeddings.

As an alternative to the original SC, connectome embeddings (CEs) can generate node embeddings that capture also higher order topological features of the structural layout (Rosenthal et al., 2018). The idea of such a graph embedding is to represent each node in the graph by a *M*-dimensional feature vector. This technique is originally inspired by the word2vec algorithm introduced by Mikolov, Sutskever, Chen, Corrado, and Dean (2013) who proposed a technique to learn vector-valued representations for words in a text which preserve linguistic regularities in their embedding space. Similarly the node2vec algorithm can be used to embed vertices of a graph into a subspace where similar embeddings capture the *k*-step (*k* = 1, 2, …, *K*) relation between the vertices and their *k*-step neighbors (Grover & Leskovec, 2016; Rosenthal et al., 2018). We used this technique to embed each brain region *n* in the SC graph into a 64-dimensional vector representation. We therefore employed the gensim python package (Řehůřek & Sojka, 2010) using the skip-gram model to learn the node representations (Mikolov et al., 2013). Briefly, in this context the idea of the skip-gram model is to predict from a target node in a network its neighboring nodes, whereby a sequence of neighboring nodes is created by performing a biased random walk on the structural graph (Grover & Leskovec, 2016). To generate the node sequences, in total 100 random walks were performed for each node with walk a length of 80 nodes. The return parameter of the random walk was set to *p* = 2 and the in-out parameter to *q* = 1. The similarity between the *N* brain regions in their embedding space was computed using the Pearson correlation coefficient, yielding a connectivity matrix denoted with **A**_*CE*_ ∈ ℝ^*N*×*N*^. As illustrated in Figure 3B, the embeddings could yield meaningful representations that revealed long-range connections between regions that were not present in the original SC (Rosenthal et al., 2018).

#### Adaptive adjacency matrix.

So far the spatial layout of the brain graph has been represented with the help of the orthogonal eigenbasis system **U** of the transition operator proportional to the random walk Laplacian. This presupposed a thorough knowledge about the spatial structure of the underlying brain network that entered the related adjacency matrix. Remember that the weights of the adjacency matrix were deduced from DTI measurements based on SC or their CE similarity. However, there may exist hidden activity correlations that are not represented in the original adjacency matrix used to construct the random walk Laplacian. Hence, one may wish to introduce an additional self-adaptive, normalized adjacency matrix **A**_*Adap*_ ∈ ℝ^*N*×*N*^ (Wu et al., 2019). The latter has been constructed as a matrix of trainable weights **V**_*Adap*_ ∈ ℝ^*N*×*N*^, which were at first initialized as zero and then again optimized via gradient descent (Kingma & Ba, 2014). Inspired by the study of Wu et al. (2019), a normalized self-adaptive adjacency matrix was computed as:

$$A_{\text{Adap}}=\frac{\sigma \left(V_{\text{Adap}}\right)}{N}$$ (10)

The transformation function *σ*(·) ≡ tanh(·) confined the adaptive weights to the range [−1, 1], which then were normalized by the number of nodes *N* in the network. This self-adaptive adjacency matrix can help to uncover any hidden, still unknown dependencies between ROIs of a given graph structure. Thus it may extend any graph diffusion convolution layer to yield its output activity as:

$$h_{t}^{\left(q\right)}=\sigma \left(y_{t}^{\left(q\right)}\right)=\sum_{k=0}^{K}\left[\theta_{k}^{\left(q\right)}T^{k}+\theta_{k}^{\left(q\right),\text{Adap}}{\left(A_{\text{Adap}}\right)}^{k}\right]x_{t}^{\left(m\right)}$$ (11)

Note that the normalized self-adaptive adjacency matrix **A**_*Adap*_ may be considered as an additional transition operator here. In an attempt to decouple the temporal processing from any underlying spatial layout of the graph connectivity, the first term within parentheses may be skipped and the self-adaptive adjacency matrix may possibly identify the underlying graph structure from the data alone. This may be applicable to situations where no predefined graph structure is known or involved. The output of the *q*-th convolution channel can in this case be obtained with:

$$h_{t}^{\left(q\right)}=\sigma \left(y_{t}^{\left(q\right)}\right)=\sigma \left(\sum_{k=0}^{K}\theta_{k}^{\left(q\right),\text{Adap}}{\left(A_{\text{Adap}}\right)}^{k}x_{t}^{\left(m\right)}\right)$$ (12)

### Temporal Dependencies

#### Recurrent neural networks.

**Figure 8.**
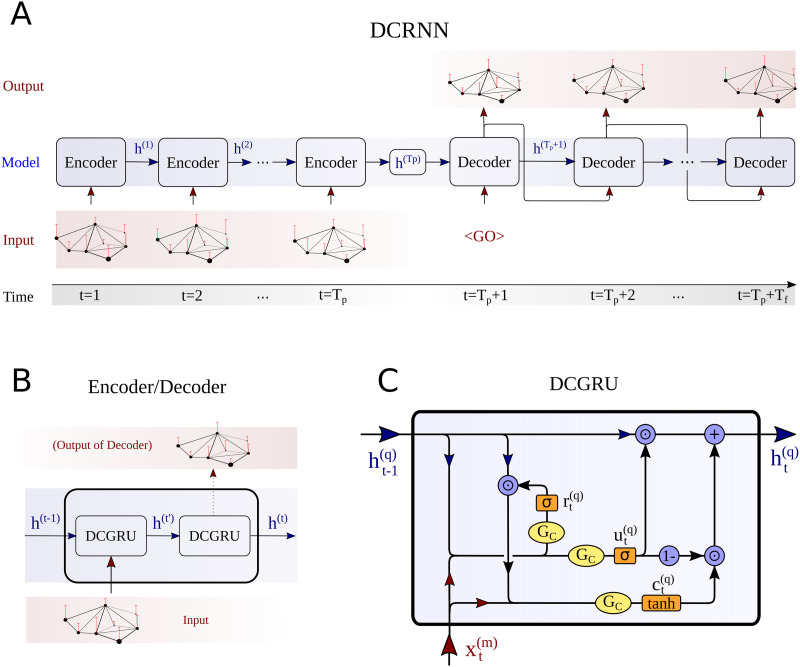
(A) Overview of the complete DCRNN model. The RNN architecture consists of an encoder and decoder, which recursively process the graph structured signals. The encoder receives a sequence of inputs [**x**^(1)^, …, **x**^(*T*_*p*_)^] and iteratively updates the hidden state **h**^(*t*)^. The final state of the encoder **h**^(*T*_*p*_)^ is passed to the decoder branch, which then recursively predicts the output sequence of future signals [**x**^(*T*_*p*_+1)^, …, **x**^(*T*_*p*_+*T*_*f*_)^]. The encoder, as well as the decoder (B) consists of multiple diffusion convolution gated recurrent unit cells (DCGRU). The first DCGRU cell receives the input graph signal and then passes its hidden state to the subsequent cell. During decoding, the final cell of the decoder then generates the predictions for the signal. For testing and validation, the decoder uses its own prediction as input for generating the subsequent prediction. The first input of the decoding branch (< *GO* > symbol) is simply a vector of zeros. The processing steps in an individual DCGRU cell are shown in (C). The input $x_{t}^{\left(m\right)}$, as well as the previous hidden state $h_{t-1}^{\left(q\right)}$ are concatenated and passed to the reset gate $r_{t}^{\left(q\right)}$, as well as to the update gate $u_{t}^{\left(q\right)}$. The reset gate $r_{t}^{\left(q\right)}$ determines the proportion of $h_{t-1}^{\left(q\right)}$, which enters $c_{t}^{\left(q\right)}$, together with input $x_{t}^{\left(m\right)}$. Then the hidden state $h_{t-1}^{\left(q\right)}$ is updated by $c_{t}^{\left(q\right)}$, whereby the amount of new information is controlled by $u_{t}^{\left(q\right)}$.

In the DCRNN model, the temporal variations of the signal $x_{t}^{\left(m\right)}$ ∈ ℝ^*N*^ in *N* brain regions at *T*_*p*_ past time points were explored with sequence-to-sequence learning in RNNs (Sutskever et al., 2014), where an encoder network compresses the information into a compact new representation. The latter is fed into a decoding network, which generates predictions of the graph signal at *T*_*f*_ future time points representing the intended prediction horizon, as illustrated in Figure 8A.

Given that the graph convolution operation accounts for the spatial layout of graph structure at any time point *t*, temporal dynamics on the graph can be modeled in the DCRNN via GRUs (Chung et al., 2014). The idea is to replace convolution operations in the spatial domain by corresponding matrix multiplications in the conjugate spatial-frequency domain employing the diffusion convolution operator. This leads to the diffusion convolution gated recurrent unit (DCGRU) (Y. Li et al., 2018):

$$\begin{array}{l} r_{t}^{\left(q\right)}=\sigma \left({𝒢}_{C}\left(\Theta_{r}^{\left(q\right)},\left[x_{t}^{\left(m\right)}\|h_{t-1}^{\left(q\right)}\right]\right)+b_{r}\right)\\ u_{t}^{\left(q\right)}=\sigma \left({𝒢}_{C}\left(\Theta_{u}^{\left(q\right)},\left[x_{t}^{\left(m\right)}\|h_{t-1}^{\left(q\right)}\right]\right)+b_{u}\right)\\ c_{t}^{\left(q\right)}=\tanh\left({𝒢}_{C}\left(\Theta_{c}^{\left(q\right)},\left[x_{t}^{\left(m\right)}\|\left(r_{t}^{\left(q\right)}⊙h_{t-1}^{\left(q\right)}\right)\right]\right)+b_{c}\right)\\ h_{t}^{\left(q\right)}=u_{t}^{\left(q\right)}⊙h_{t-1}^{\left(q\right)}+\left(1-u_{t}^{\left(q\right)}\right)⊙c_{t}^{\left(q\right)} \end{array}$$ (13)

where $x_{t}^{\left(m\right)}$, $h_{t}^{\left(q\right)}$ denote the *m*-th input and *q*-th output graph signal feature component of the GRU, respectively, at time *t*, and [$x_{t}^{\left(m\right)}$ ‖ $h_{t-1}^{\left(q\right)}$] denotes their concatenation. Also $r_{t}^{\left(q\right)}$, $u_{t}^{\left(q\right)}$ represent reset and update gates at time *t*, and **b**_*r*_, **b**_*u*_, **b**_*c*_, respectively, denote bias terms. Furthermore, $\Theta_{r}^{\left(q\right)}$, $\Theta_{u}^{\left(q\right)}$, $\Theta_{c}^{\left(q\right)}$ denote the parameter sets of the corresponding filters. An illustration of the complete sequence-to-sequence architecture incorporating DCGRU cells is provided in Figure 8.

#### WaveNets.

**Figure 9.**
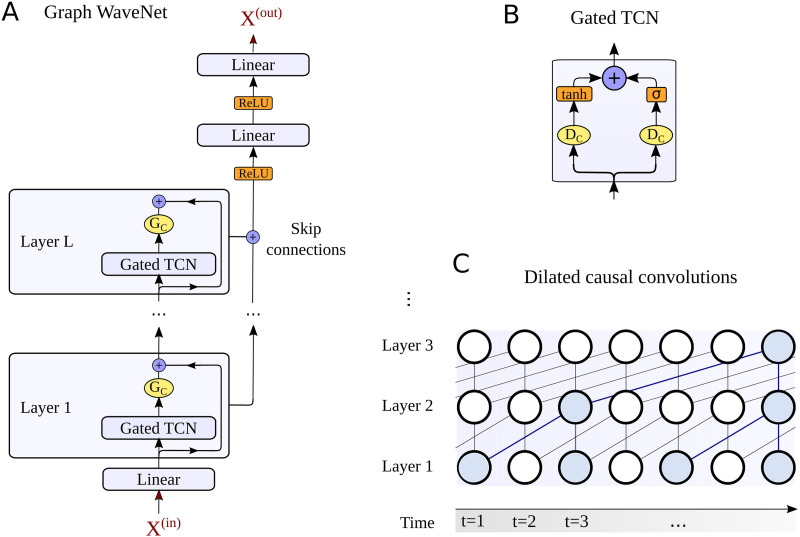
(A) An overview of the complete GWN model. The GWN model consists of *L* layers. For the temporal modeling, the GWN applies first the gated TCN mechanism and then for the spatial aspects utilizes graph convolution operations (𝒢_*C*_) in each layer. Each layer additionally incorporates residual connections to stabilize the gradient during learning (He et al., 2016). The information in each layer is combined by using skip connections, and the final predictions are generated by passing the output of the skip connections through two fully connected layers. The gated temporal convolution network (TCN) mechanism (B) applies a dilated causal convolution (𝒟_*C*_) in combination with a tanh(·) and a *σ*(·) activation function to control the information flow. (C) The dilated causal convolutions are illustrated. In each layer a temporal convolution is applied whereby the dilation factor can be increased in subsequent layers. These dilations lead to exponentially growing receptive fields for neurons in higher layers. The receptive field of a neuron in layer is highlighted in blue.

Rather than incorporating diffusion convolution layers into RNNs, dilated causal convolution (DCC) layers (van den Oord et al., 2016) have been instead employed in the GWN architecture (Wu et al., 2019). The full GWN model is illustrated in Figure 9. The DCC was defined through a dilated causal convolution operator 𝒟_*C*_:

$${𝒟}_{C}\left(\Theta_{t}^{\left(q\right)},x_{t}^{\left(m\right)}\right)=\sum_{r=0}^{R-1}\Theta_{r}^{\left(q\right)}x_{t-d\cdot r}^{\left(m\right)}$$ (14)

whereby *d* denoted the dilation factor and $\Theta_{t}^{\left(q\right)}$ represented the filter kernel. DCC could be implemented by sliding over the input time series $x_{t}^{\left(m\right)}$ while skipping input values while, from layer to layer, increasing step size *d* · *r*. This procedure leads to an exponential growth of the receptive field with increasing layer depth as is schematically illustrated in Figure 9C. The information flow was controlled by a gated temporal convolution network (TCN) as shown in Figure 9B, which is obtained as:

$$h_{t}^{\left(q\right)}=\tanh\left({𝒟}_{C}\left(\Theta_{1}^{\left(q\right)},x_{t}^{\left(m\right)}+b_{1}\right)\right)⊙\sigma \left({𝒟}_{C}\left(\Theta_{2}^{\left(q\right)},x_{t}^{\left(m\right)}+b_{2}\right)\right)$$ (15)

Here tanh(·) denotes the output activation function, and $\Theta_{1}^{\left(q\right)}$, $\Theta_{2}^{\left(q\right)}$, and **b**_1_, **b**_2_ represent the convolution filers and bias terms, respectively. Further, 𝒟_*C*_ represents the causal convolution operator, ⊙ the Hadamard product and *σ*(·) denotes the logistic function, which controls the information passed to the next layer. To achieve large receptive fields, the layers in a WN architecture are organized in blocks, whereby in each block the dilation factor *d* is doubled with *d* = 1, 2, 4, … up to a certain limit and then repeated in the same manner in the next block (van den Oord et al., 2016). After each such dilated convolution layer, a diffusion convolution layer 𝒢_*C*_ (Equation 9) is subsequently applied to account for the spatial dependencies, as illustrated in Figure 9A.

#### Temporal relevance.

**Figure 10.**
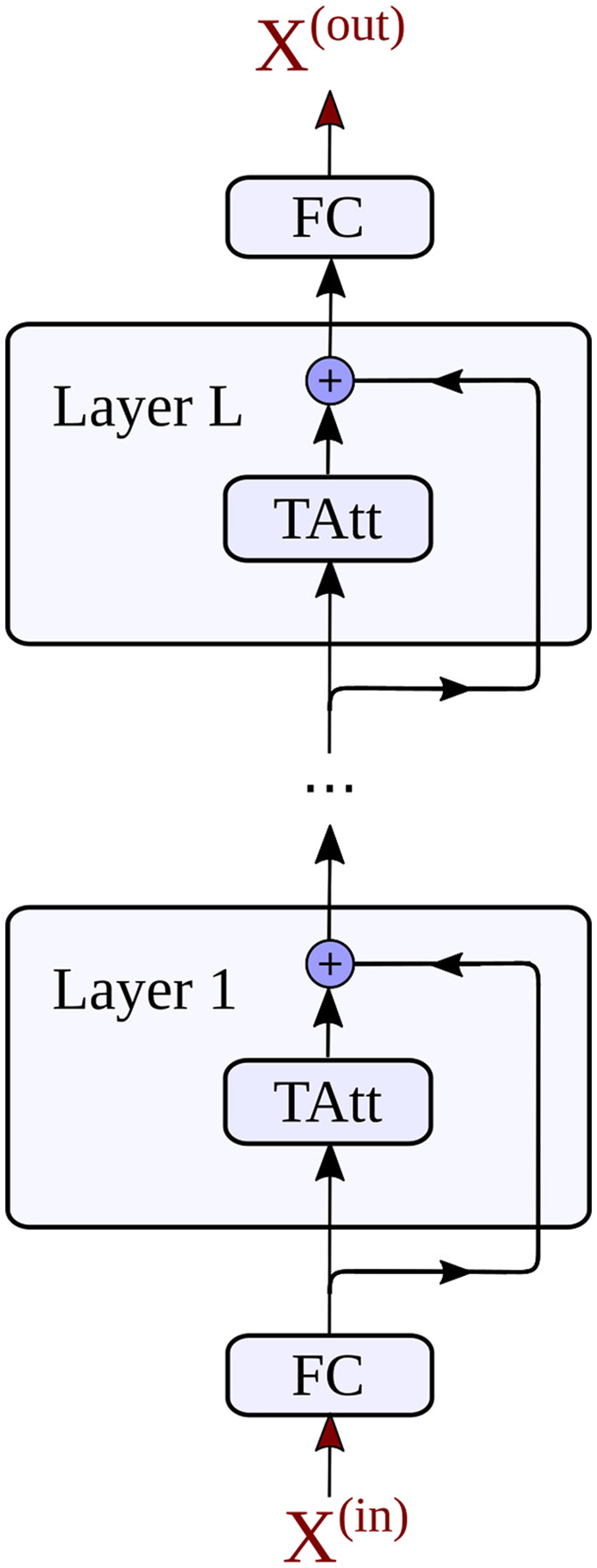
An overview over the temporal relevance or attention model. The single feature input *X*^(*in*)^, representing the BOLD signal, is first projected by a fully connected layer onto *M* output features. The temporal relevance scores are computed in each of the *L* attention layers, and to further account for vanishing gradients, additional residual connects are incorporated (He et al., 2016). The output of the final layer *L* is then projected back onto a single feature, representing the predicted neural signal.

Yet another approach to solve spatiotemporal time series prediction problems considers attention mechanisms in spatial and temporal domains to capture dynamic correlations (Vaswani et al., 2017; Zheng et al., 2020). In this study, we therefore additionally explore nonlinear temporal correlations via a temporal relevance mechanism for modeling temporal fluctuations in the BOLD signal. Let the temporal state of the brain network be represented by the multivariate signal tensor 𝒳 ∈ ℝ^*N*×*T*×*M*^ such that the temporal states of any node *n* be collected in the signal matrix 𝒳_*n*::_ ≡ **X**^(*n*)^ ∈ ℝ^*T*×*M*^. The activity at any node *n* and at any time *t* was then represented by the tube fibers ${\tilde{x}}_{t}^{\left(n\right)}$ ∈ ℝ^*M*^, where *M* denoted the number of features characterizing the node activity. Temporal correlations between different node states could be estimated by filtering the multivariate signals in a cascade of temporal relevance blocks, as illustrated in Figure 10. The queries and keys are computed from the input in the *ℓ*-th block at time point *t* with a simple nonlinear transformation *g*_*r*_(${\tilde{x}}_{t}^{\left(n\right)}$) = ReLU(**W**_*r*_${\tilde{x}}_{t}^{\left(n\right)}$ + **b**_*r*_) with parameters **W**_*r*_ ∈ ℝ^*D*×*M*^ and **b**_*r*_ ∈ ℝ^*D*^. For any node *n* and any time point *t*_*i*_ the relevance of its states ${\tilde{x}}_{t_{i}}^{\left(n\right)}$ at time points *t*_*j*_ < *t*_*i*_ with respect to the considered state ${\tilde{x}}_{t_{j}}^{\left(n\right)}$ could then be assessed by computing the inner product between the queries and keys:

$$\delta_{t_{i},t_{j}}^{\left(n\right)}=\frac{\left(g_{r}\left({\tilde{x}}_{t_{i}}^{\left(n\right)}\right)\right)\cdot {\left(g_{r}\left({\tilde{x}}_{t_{j}}^{\left(n\right)}\right)\right)}^{T}}{\sqrt{D}}.$$ (16)

A normalized temporal relevance score ${\hat{\delta }}_{\left(t_{i},t_{j}\right)}^{\left(n\right)}$ could then be computed according to:

$${\hat{\delta }}_{t_{i},t_{j}}^{\left(n\right)}=\frac{\exp\left(\delta_{t_{i},t_{j}}^{\left(n\right)}\right)}{\sum_{t_{j}<t_{i}}\exp\left(\delta_{t_{i},t_{j}}^{\left(n\right)}\right)}$$ (17)

Finally, *t*_*j*_ < *t*_*i*_, *j* ∈ {1, …, *T*_*p*_} denoted a set of time steps before time point *t*_*i*_. After computing the temporal relevance score ${\hat{\delta }}_{t_{i},t_{j}}^{\left(n\right)}$, the hidden state of node *n* at time *t*_*i*_ could be derived as:

$${\tilde{h}}_{t_{i}}=g_{r}\left(\sum_{t_{j}<t_{i}}{\hat{\delta }}_{t_{i},t_{j}}^{\left(n\right)}\cdot g_{r}\left({\tilde{x}}_{t_{j}}^{\left(n\right)}\right)\right)$$ (18)

whereby *g*_*r*_(·) denotes a nonlinear projection again. Note that all parameters **W**_*r*_ and **b**_*r*_ to be learned were shared across all nodes and time steps. In total, *L* layers of temporal attention mechanisms were stacked to generate a final prediction for the BOLD signal. After each layer batch, normalization was applied and additionally residual connections were incorporated to stabilize the gradient (He et al., 2016).

### Model Training

In this section we outline the training procedures that were used for the different neural network models to learn the temporal and spatial dynamics in the BOLD signal. Before training, the fMRI data of each session was linearly scaled between 0 and 1, to get gradients of a small magnitude during the backpropagation learning, which facilitates the fine-tuning of the learning rate. For all models, the mean absolute error (MAE) was used as an objective function to quantify the overall difference between the true BOLD signal **x**^(*t*)^ and predicted signal $\hat{x}$^(*t*)^ in all *N* brain regions:

$$MAE\left(x,\hat{x}\right)=\frac{1}{N}\sum_{n=1}^{N}\frac{1}{T_{f}}\sum_{t=1}^{T_{f}}\left|x_{n}^{\left(t\right)}-{\hat{x}}_{n}^{\left(t\right)}\right|$$ (19)

#### DCRNN.

The DCRNN model, based on an RNN architecture, was trained with backpropagation through time (Werbos, 1990), with the objective to maximize the likelihood of generating the target time series. To additionally account for a mismatch between training and testing distributions of stimuli, a scheduled sampling strategy was used (Bengio, Vinyals, Jaitly, & Shazeer, 2015). The probability of using a true label as a decoder input decayed according to:

$$\epsilon \left(i\right)=\frac{\tau }{\tau +\exp\left(i/\tau \right)}\in \left(0,1\right)$$ (20)

with *τ* > 0 the decay parameter and *i* ∈ ℕ counting the iterations. During supervised learning, instances to be predicted were, of course, known. For this optimization problem, the Adam algorithm (Kingma & Ba, 2014) was employed, and the model was trained for 70 epochs on minibatches of 16 training samples. To further improve convergence, an annealing learning rate was used, initialized as *η* = 0.1, and decreased by a factor of 0.1 at epochs 20, 40, and 60, or if the validation error did not improve for more than 10 epochs. Before lowering the learning rate, the weights with lowest validation error were restored, in order to avoid getting stuck in local optima. For the training dataset including only 10 subjects used in the Model Accuracy and Network Scaling section, the number of training epochs was increased to 140 and the learning rate decay applied at epochs 40, 80, and 120. The influence of the DCRNN model hyperparameters are discussed in Supporting Information I (Figure S1) and were chosen to yield a reasonable trade-off between accuracy and computational requirements. The encoder and decoder of the sequence-to-sequence architecture consist to two diffusion convolution GRU layers each, and the hidden state size was set to 64. The computations were performed on a Nvidia RTX 2080 Ti GPU, running on a desktop PC with an Intel(R) Core(TM) i7-9800X CPU under Linux Ubuntu 20.04. With this setup one epoch on the dataset including 25 subjects and predicting the activity within one hemisphere including 180 ROIs took approximately 3.4 minutes. To have error values of a magnitudes that are easier to interpret, for the evaluations in the Results section the whole dataset was rescaled to zero mean and unit variance after the training of all STGNN models was finished.

#### GWN.

The GWN model was also trained incorporating the Adam optimizer (Kingma & Ba, 2014) to minimize the MAE defined in Equation 19. For the GWN model, it was sufficient to train it 30 epochs with a batch size of eight, thereby initializing the learning rate with *η* = 0.0001 and decreasing it by a factor of 0.1 at epochs 10 and 20. For the 10 subject dataset, the number of epochs was also increased to 60 and the learning rate decay adapted to epochs 20 and 40 correspondingly. The influence of the hyperparameters of the GWN is evaluated in Supporting Information I (Figure S2). A good trade-off between model accuracy and complexity could be found using 32 neurons. The number of layers per block were defined as 2 with a total number of 12 blocks. With this setup, one epoch on the 25 subjects’ dataset including 180 ROIs took around 12.2 minutes.

#### TAtt.

The TAtt model was trained using the Adam optimizer (Kingma & Ba, 2014) for in total 40 epochs, minimizing the MAE defined in Equation 19, using a batch size of 16. The learning rate was initialized with *η* = 0.1 and decreased by a factor of 0.1 at epochs 10, 20, and 30. The influence of the hyperparmeters is evaluated in Supporting Information I (Figure S3). The number of neurons in the temporal attentions were set to 32 thereby using four attention heads in the four TAtt layers. With this setup of hyperparameters one epoch of the TAtt model took around 7.7 minutes.

### Vector Autoregressive Model

Granger causality (Granger, 1969) is currently most often based on linear vector autoregressive (VAR) models for stochastic time series data. Therefore we compare our GNN-based approach with a VAR model, as implemented in the multivariate Granger causality (MVGC) toolbox (Barnett & Seth, 2013). An autoregressive process is based on the idea that a signal *x*^(*t*)^ can be described as a linear superposition of the first *T*_*p*_ of its lagged values (Luetkepohl, 2005):

$$x^{\left(t\right)}=\beta +\alpha_{1}x^{\left(t-1\right)}+\alpha_{2}x^{\left(t-2\right)}+\cdots +\alpha_{p}x^{\left(t-T_{p}\right)}+u^{\left(t\right)}$$ (21)

with coefficients or weights *α*_1_, …, *α*_*p*_, an intercept *β* and the error term *u*^(*t*)^. This univariate formulation can be then extended to a multivariate VAR model including *N* time series **x**^(*t*)^ = [$x_{1}^{\left(t\right)}$, …, $x_{N}^{\left(t\right)}$] (Luetkepohl, 2005):

$$x^{\left(t\right)}=b+A_{1}x^{\left(t-1\right)}+A_{2}x^{\left(t-2\right)}+\cdots +A_{p}x^{\left(t-T_{p}\right)}+u^{\left(t\right)}$$ (22)

whereby the coefficients are now collected in matrices **A** ∈ ℝ^*N*×*N*^, and intercepts and errors are characterized by vectors **b** ∈ ℝ^*N*^ and **u**^(*t*)^ ∈ ℝ^*N*^, respectively. In our study, the multivariate time series **x**^(*t*)^ reflect the BOLD signal strength in the *N* brain regions, sampled at different timesteps *t*.

To estimate parameters of the VAR model, we used the ordinary least squares fit provided in the MVGC toolbox (Barnett & Seth, 2013). As outlined in the Data Description section, we used the first 80% of the data from each fMRI session to fit the model. Then for the comparison to the GNN approaches in the Model Accuracy and Network Scaling section, we tested the model order *p* in steps of five with *p* = 5, 10, …, *T*_*p*_ and chose the model with highest accuracy individually for each dataset. To check for stationarity of the signals, an augmented Dickey-Fuller test for unit roots was applied to the BOLD time courses (Hamilton, 1994; MacKinnon, 1994), using a *p* value of *p* < 0.01. For the 25 subject dataset, around 10.0% of the BOLD time courses do not fulfill the stationarity criteria of the augmented Dickey-Fuller test (*p* > 0.01) when using such a high lag order of *T*_*p*_ = 60. But as the objective criterion of the evaluation in the Model Accuracy and Network Scaling section was to assess the capabilities of the models to predict empirically observed neural activity patterns, we chose the VAR model with best prediction accuracy for comparisons with the GNNs.

## ACKNOWLEDGMENTS

Data were provided by the Human Connectome Project, WU-Minn Consortium (Principal Investigators: David Van Essen and Kamil Ugurbil; 1U54MH091657) funded by the 16 NIH Institutes and Centers that support the NIH Blueprint for Neuroscience Research, and by the McDonnell Center for Systems Neuroscience at Washington University.

## SUPPORTING INFORMATION

Supporting information for this article is available at https://doi.org/10.1162/netn_a_00252. A demo version for MRI data preparation and training the DCRNN model is provided at https://github.com/simonvino/DCRNN_brain_connectivity. In addition, a demo version for the GWN model is provided at https://github.com/simonvino/GraphWaveNet_brain_connectivity. Preprocessed HCP data is publicly available under: https://db.humanconnectome.org.

## AUTHOR CONTRIBUTIONS

Simon Wein: Conceptualization; Investigation; Methodology; Writing – original draft. Alina Schüller: Investigation; Writing – review & editing. Ana Maria Tomé: Validation; Writing – review & editing. Wilhelm M. Malloni: Validation; Writing – review & editing. Mark W. Greenlee: Supervision; Writing – review & editing. Elmar W. Lang: Methodology; Supervision; Writing – original draft; Writing – review & editing.

## FUNDING INFORMATION

Mark W. Greenlee, Deutsche Forschungsgemeinschaft (https://dx.doi.org/10.13039/501100001659), Award ID: GR988/25-1. Mark W. Greenlee, Deutsche Forschungsgemeinschaft (https://dx.doi.org/10.13039/501100001659), Award ID: ISNT89/393-1.

## TECHNICAL TERMS

Graph neural network: A type of artificial neural network that is used to extract features from data with graph-like geometries.
Convolution: Denotes in the context of artificial neural networks a discrete mathematical convolution operation of the input of a neural network layer with a localized filter kernel, whereby the filter kernels are learned during neural network training.
Deep learning: Learning in an artificial neural network that includes multiple hidden representations of the neural network input.
Graph convolutions: Denotes a discrete convolution operation generalized to the non-Euclidean geometry of graphs. Similar to classical convolution operations in CNNs, they entail principles like sparse interactions and parameter sharing.
Exploding gradients: Describes the phenomenon when the gradients of the neural network model weights grow excessively during backpropagation learning.
Vanishing gradients: Describes the phenomenon when the gradients of the neural network model weights become very small during backpropagation learning.
Receptive field: Denotes the area in the artificial neural network input a neuron is (possibly via multiple layers) connected to.
Spatial information exchange: Describes in the context of spatiotemporal graph signal processing the exchange of information along the edges of a graph signal.
Spatial autocorrelations: The phenomenon in neuroscience that activity in nearby areas tends to be more strongly correlated than in more distant areas.
Temporal autocorrelations: Characterizes the smoothness of the memory of neural activity time courses in different brain regions.
