# Supplementary material for: Forecasting brain activity based on models of spatiotemporal brain dynamics: A comparison of graph neural network architectures
Source: Netw Neurosci. 2022 Jul 1;6(3):665–701. doi: 10.1162/netn_a_00252 (PMC9810370; doi:10.1162/netn_a_00252)
Supplement: Supplementary file 1 [file netn-06-665-s001.pdf]

Wein, S. Schüller, A., Tomé, A. M., Malloni, W. M., Greenlee, M. W. & Lang, E. W. (2022). Supporting information for "Forecasting brain activity based on models of spatio-temporal brain dynamics: a comparison of graph neural network architectures. *Network Neuroscience,* 6(3), 665–701. https://doi.org/10.1162/netn_a_00252

Forecasting Brain Activity Based on Models of Spatio-Temporal Brain Dynamics: A Comparison of Graph Neural Network Architectures

S. Wein[1,2], A. Schüller[1], A. M. Tomé[3], W. M. Malloni[2], M. W. Greenlee[2], and E. W. Lang[1]

[1]CIML, Biophysics, University of Regensburg, Regensburg, Germany
[2]Experimental Psychology, University of Regensburg, Regensburg, Germany
[3]IEETA/DETI, Universidade de Aveiro, Aveiro, Portugal

Supplementary Information

Supplement I

In this supplement the influence of the model hyperparameters for the different neural network architectures is discussed. The hyperparameters are chosen as described in the 'Model training' section and held constant, while only the hyperparameter of interest is varied in the following evaluations. Figure S1 and S2 show that the performance of he DCRNN and GWN in general still could slightly improve with a larger number of model parameters. Because the computation time and memory requirements linearly grow with the number of parameters, we chose the model hyperparameters as described in the 'Model training' section to yield a good trade-off between model performance and computational requirements. Also the TAtt model in figure S3 shows some improvement with a larger number of parameters, however the MAE is still considerably higher compared to the RNN and WN based GNN architectures.

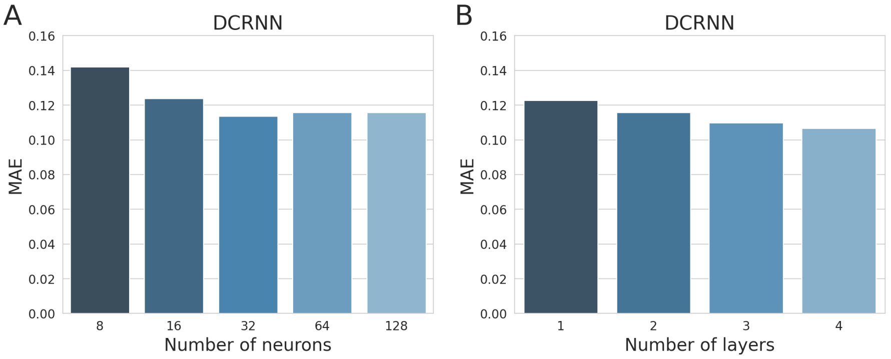

Figure S1: Here the influence of the hyperparameters on the prediction accuracy of the DCRNN is depicted. In (A) the test error is shown in dependence of the number of neurons in each layer, and in (B) the error in dependence of the number of DCGRU layers.

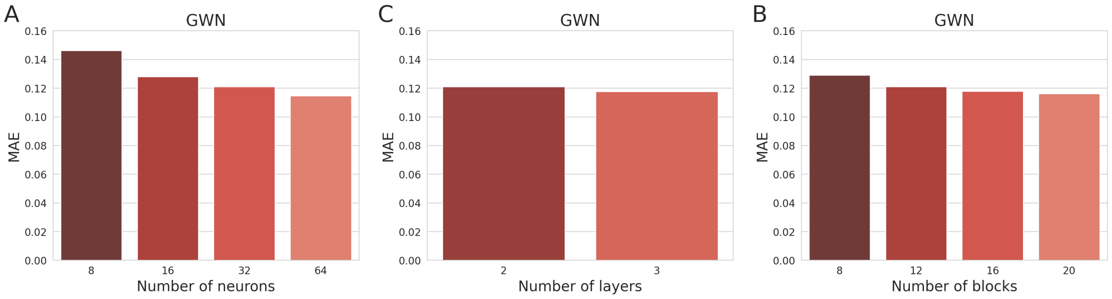

Figure S2: In this figure the influence of the GWN hyperparameters on the prediction accuracy is shown. In (A) the test error in dependence of number of neurons (or feature maps) is illustrated. Here (B) shows the influence of the number of DCC blocks used in the GWN architecture and (C) shows the impact of the number of layers per DCC block.

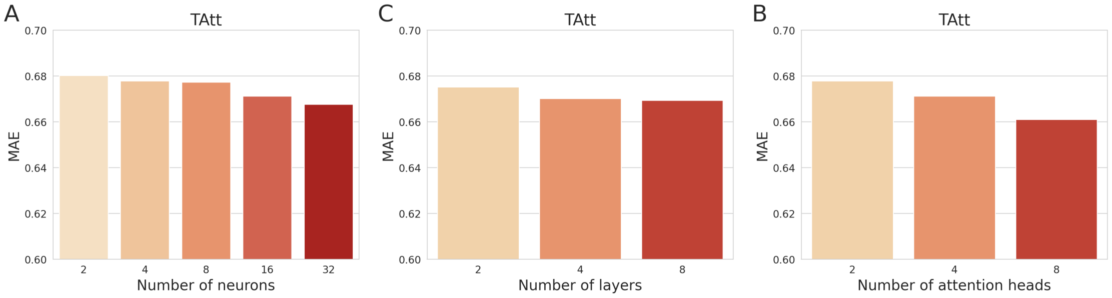

Figure S3: Here the influence of hyperparameters on the TAtt accuracy is illustrated. In (A) the test MAE in dependence of the number of neurons used in each TAtt mechanism is shown. In (B) the influence of the number of TAtt layers is depicted and (C) illustrates the impact of number of attention heads incorporated.

Supplement II

Table 1: List of ROIs involved in visual processing based on the multi-modal parcellation proposed by Glasser et al. [1]. The table shows the index of the region in the atlas for the right/left hemisphere including the name of the region.

Index	Name
1/181	V1
2/182	MST
3/183	V6
4/184	V2
5/185	V3
6/186	V4
7/187	V8
13/193	V3A
16/196	V7
19/199	V3B
20/200	LO1
21/201	LO2
22/202	PIT
23/203	MT
152/332	V6A
153/333	VMV1
154/334	VMV3
156/336	V4t
158/338	V3CD
159/339	LO3
160/340	VMV2
163/343	VVC

Supplement III

In this supplement the different spatial and temporal modeling approaches are evaluated using the scale-free R-squared (R^2) measure and the similarity of predicted connectivity states. The R^2 measure can be obtained with:

$$R^2(\mathbf{x}, \hat{\mathbf{x}}) = 1 - \frac{\sum_{n,t}(x_n^{(t)} - \hat{x}_n^{(t)})^2}{\sum_{n,t}(x_n^{(t)} - \bar{x}_n^{(t)})^2} \tag{1}$$

where $x_n^{(t)}$ represents the true BOLD signal in brain region n at timestep t. Correspondingly $\bar{x}_n^{(t)}$ denotes the average BOLD signal across all N regions and T_f timepoints, and $\hat{x}_n^{(t)}$ indicates the predicted signal of the respective model. Using this scale-free measure the evaluations of the section 'Comparison of GNN architectures' are replicated in figure S4. To test the significance also based on this measure, the R^2 values were computed for the individual subjects, and by applying a paired t-test the RNN and WN model both outperformed the TAtt model with a p-value of $p \leq 0.0001$ (Cohen's $d \gg 1$) based on this measure. Also the impact of structural modeling showed to be significant with $p \leq 0.0001$ (Cohen's $d > 1$) for both STGNN models.

In addition, we evaluated the similarity of the predicted FC states to the ground truth FC states. For this purpose we computed a FC matrix $\mathbf{A}_{FC} \in \mathbb{R}^{N \times N}$ from the true ROI timecourses $\mathbf{x} \in \mathbb{R}^{N \times T_f}$, and respectively the predicted FC matrix $\hat{\mathbf{A}}_{FC}$ from predicted timecourses $\hat{\mathbf{x}}$ based on Pearson correlation. Then we sorted the elements below the diagonal of the symmetric FC matrix $\mathbf{A}_{FC}$ into a vector $\mathbf{v} \in \mathbb{R}^{\frac{1}{2}N(N-1)}$, and defined a correlation based measure of FC similarity as:

$$r(\mathbf{v}, \hat{\mathbf{v}}) = \frac{\sum_{c=1}^{\frac{1}{2}N(N-1)}(\mathbf{v}_c - \bar{\mathbf{v}}_c)(\hat{\mathbf{v}}_c - \bar{\hat{\mathbf{v}}}_c)}{\sigma_{\mathbf{v}}\sigma_{\hat{\mathbf{v}}}} \tag{2}$$

with $\bar{\mathbf{v}}_c$ denoting the average and $\sigma_{\mathbf{v}}$ the variance of FC values. Based on this metric we replicated the comparison of spatial and temporal GNN approaches in figure S5. Also using this similarity measure, the RNN and WN model outperformed the TAtt model with $p \leq 0.0001$ (Cohen's $d \gg 1$) across subjects. The impact of structural modeling showed to be significant with $p \leq 0.0001$ (Cohen's $d > 1$) across subjects for both STGNN models. In addition the distributions of the test MAE across different subjects, timepoints and brain ROIs are visualized for the STGNNs with and without incorporating spatial modeling in figure S6.

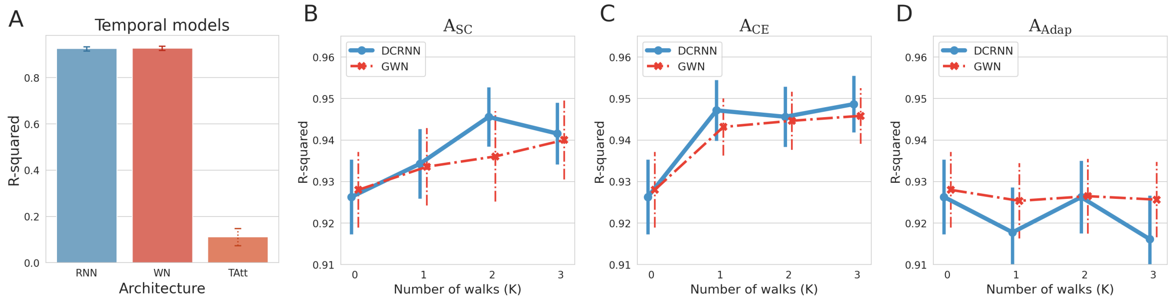

Figure S4: Figure (A) shows a comparison of different modeling strategies for temporal dynamics in the BOLD signal, comparing the $\mathbf{R}^2$ values of the recurrent neural network (RNN), the WaveNet (WN) and the temporal attention (TAtt) architecture. The errorbars represent the standard deviation of the values across subjects. Figure (B), (C) and (D) show the prediction accuracies of the DCRNN and GWN model in dependence of the walk order K. In figure (B) the $\mathbf{R}^2$ values are shown when incorporating the SC as an adjacency matrix $\mathbf{A}_{SC}$, figure (C) illustrates the test MAE when employing CEs in an adjacency matrix $\mathbf{A}_{CE}$ to define spatial relationships, and (D) displays the case when using a self-adaptive weight matrix $\mathbf{A}_{Adap}$.

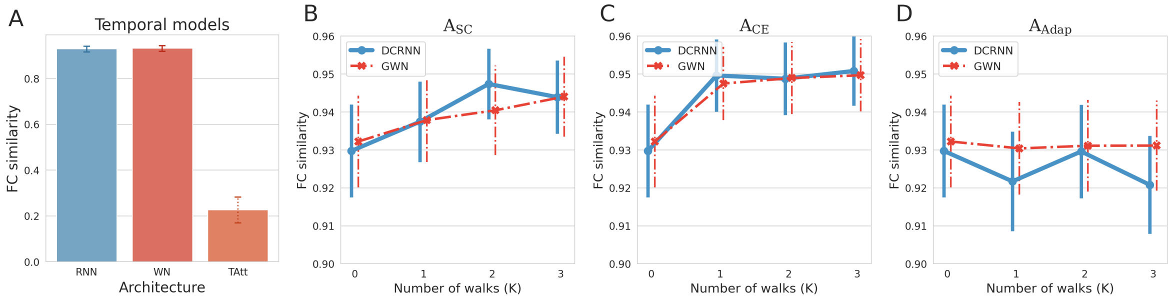

Figure S5: Figure (A) shows a comparison of different modeling strategies for temporal dynamics in the BOLD signal, comparing the FC similarities of the recurrent neural network (RNN), the WaveNet (WN) and the temporal attention (TAtt) architecture. The errorbars represent the standard deviation of the values across subjects. Figure (B), (C) and (D) show the prediction accuracies of the DCRNN and GWN model in dependence of the walk order K. In figure (B) the similarity values are shown when incorporating the SC as an adjacency matrix $\mathbf{A}_{SC}$, figure (C) illustrates the test MAE when employing CEs in an adjacency matrix $\mathbf{A}_{CE}$ to define spatial relationships, and (D) displays the case when using a self-adaptive weight matrix $\mathbf{A}_{Adap}$.

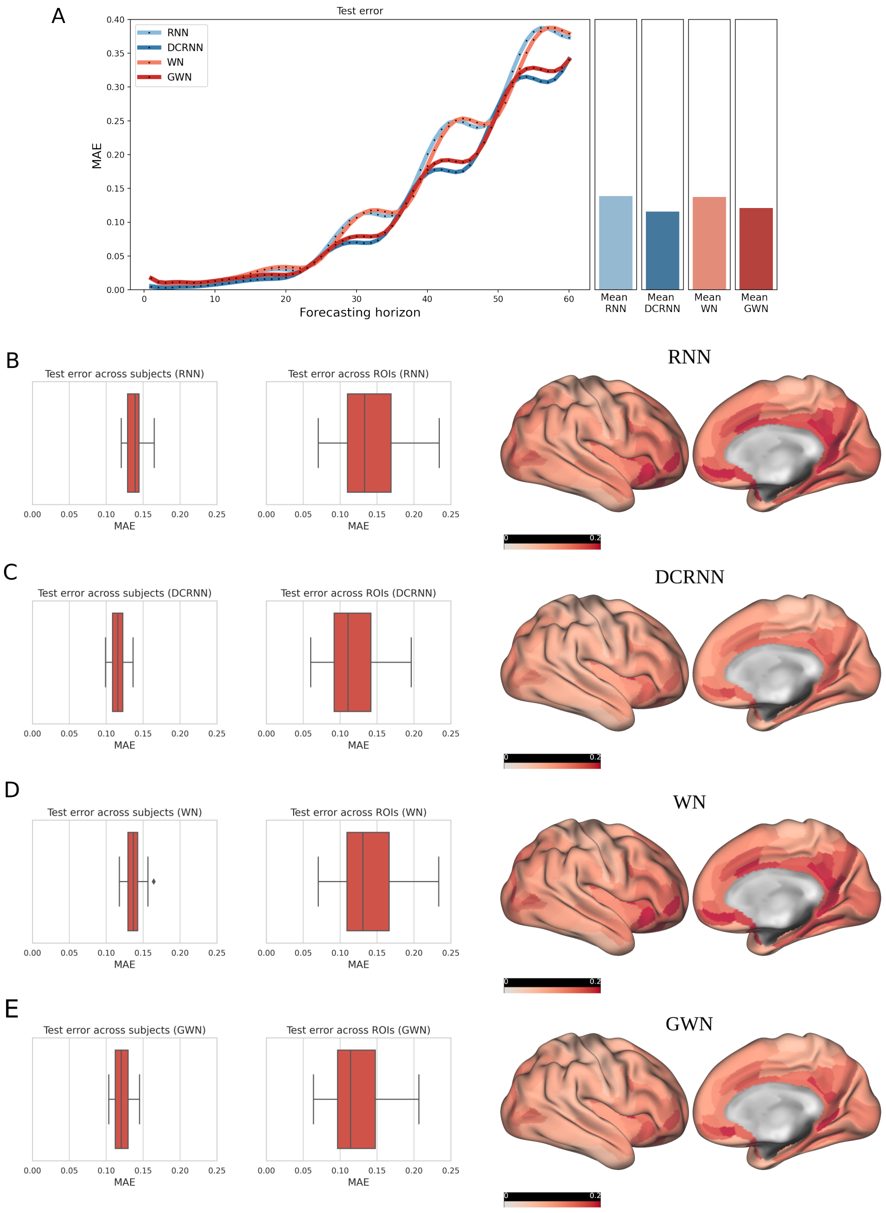

Figure S6: The distribution of the test MAE error across timepoins, subjects and ROIs is illustrated for the STGNNs, with and without incorporating spatial modeling. In (A) the prediction error in dependence of the forecasting horizon is depicted. Further (B) shows the MAE across subjects and brain regions of the RNN, visualized in boxplots on the left side. On the right side the MAEs in dependence of the brain regions were projected onto the cortical surface. The same analysis was carried out for the DCRNN, WN and GWN model in figure (C), (D) and (E) respectively.

Supplement IV

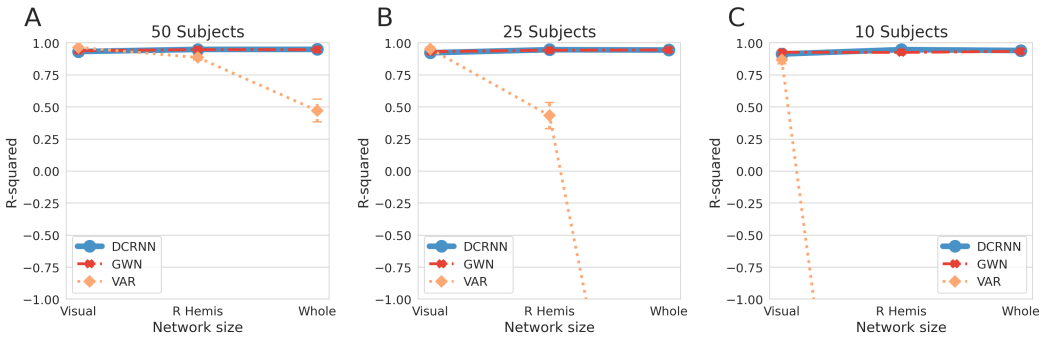

Figure S7: The figure shows a comparison of the model performances, based on the R^2 measure as defined in equation 1. The errorbars represent the standard deviation of the R^2 values across subjects. In (A) the model accuracies when using a dataset of 50 subjects are shown for the visual network, the network within the right hemisphere and the whole brain network. Figure (B) and (C) show the test performances in dependence of the network size using the 25 and 10 subject dataset, respectively. Except for the single network dataset, the improvements of accuracy of the DCRNN and GWN in comparison to the VAR model became in all cases highly significant with $p \leq 0.0001$ (Cohen's $d \gg 1$).

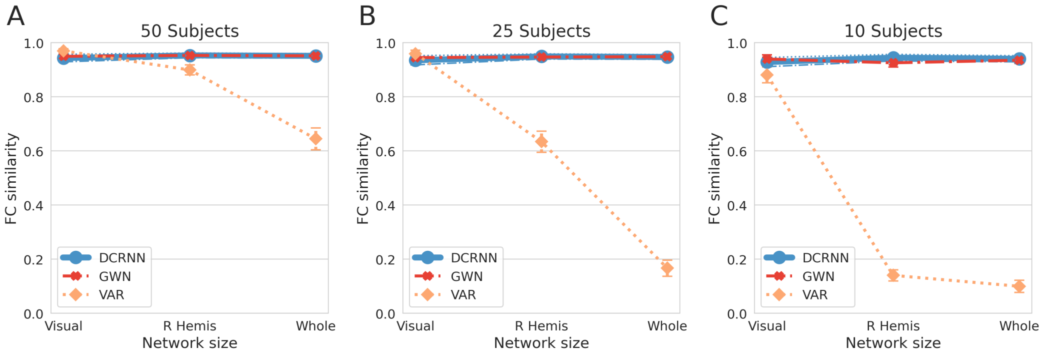

Figure S8: The figure shows a comparison of the model performances, based on the similarity of predicted FC states, as defined in equation 2. The errorbars represent the standard deviation of the values across subjects. In (A) the model accuracies when using a dataset of 50 subjects are shown for the visual network, the network within the right hemisphere and the whole brain network. Figure (B) and (C) show the test performances in dependence of the network size using the 25 and 10 subject dataset, respectively. Except for the single network dataset, the improvements of accuracy of the DCRNN and GWN in comparison to the VAR model became in all cases highly significant with $p \leq 0.0001$ (Cohen's $d \gg 1$).

Supplement V

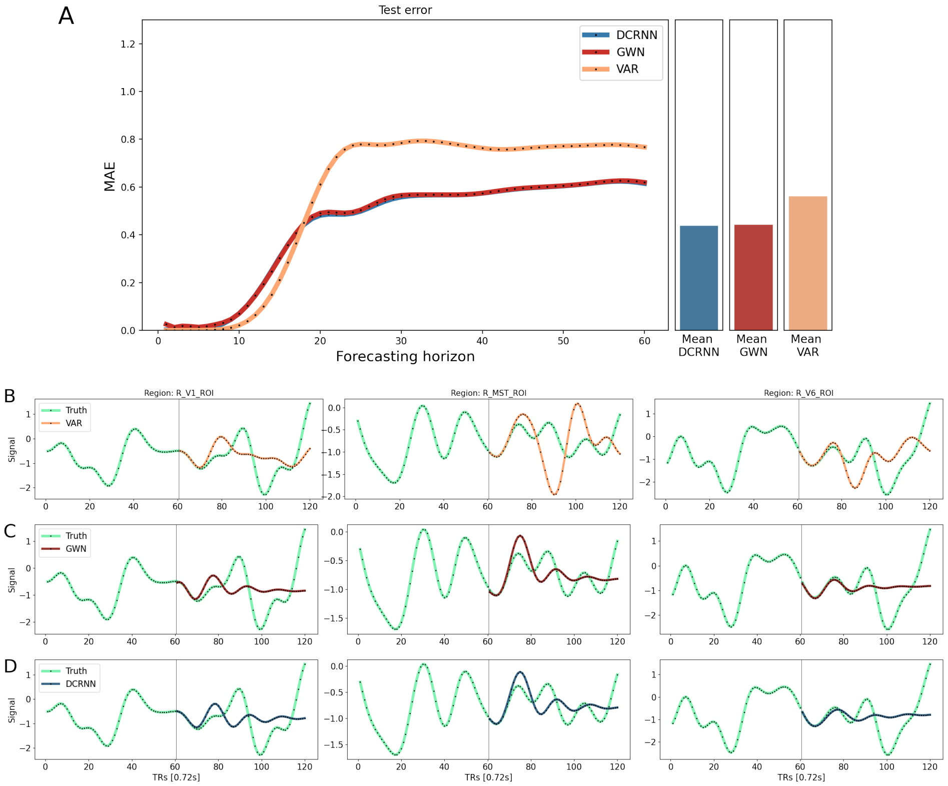

Figure S9: In this figure the prediction accuracy of the different models is presented when using a bandpass filter within the $0.01 - 0.1Hz$ frequency range for the 25 subject dataset and the brain network including the ROIs within the right hemisphere [1]. In (A) the test MAE in dependence of the forecasting horizon is shown, computed as an average across test samples and brain regions. Figure (B), (C) and (D) show examples of predictions generated by the VAR, GWN and DCRNN model respectively. The examples in this figures were chosen to be representative for the whole test set, by selecting only examples which errors maximally deviate by 0.02 from the corresponding average test MAE of the models.

Supplement VI

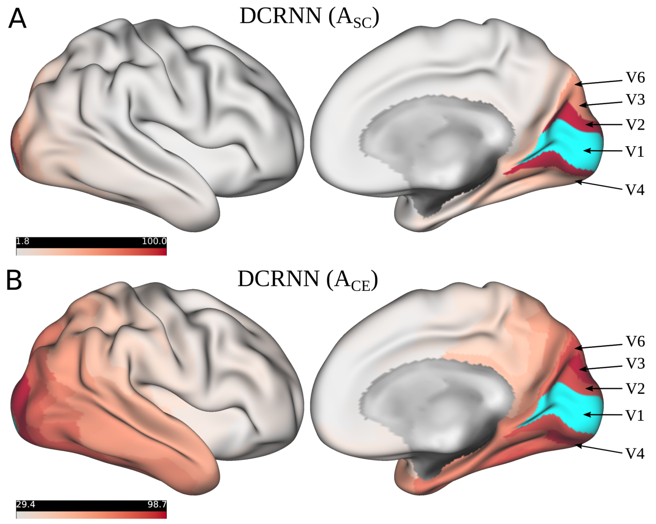

Figure S10: This figure illustrates directed spatial relations learned by the DCRNN model. Figure (A) shows the measures of influence $\mathbf{I}(n')$, derived from the DCRNN model when using the SC for information propagation and figure (B) depicts the influence when incorporating CEs for the information exchange. The values of the connectivity measures were linearly mapped between 0 and 100 and the default scaling of the color values provided by the *connectome workbench* (version 1.4.2) was used, adjusting the colormap between the $2th$ and $98th$ percentile of the values respectively.

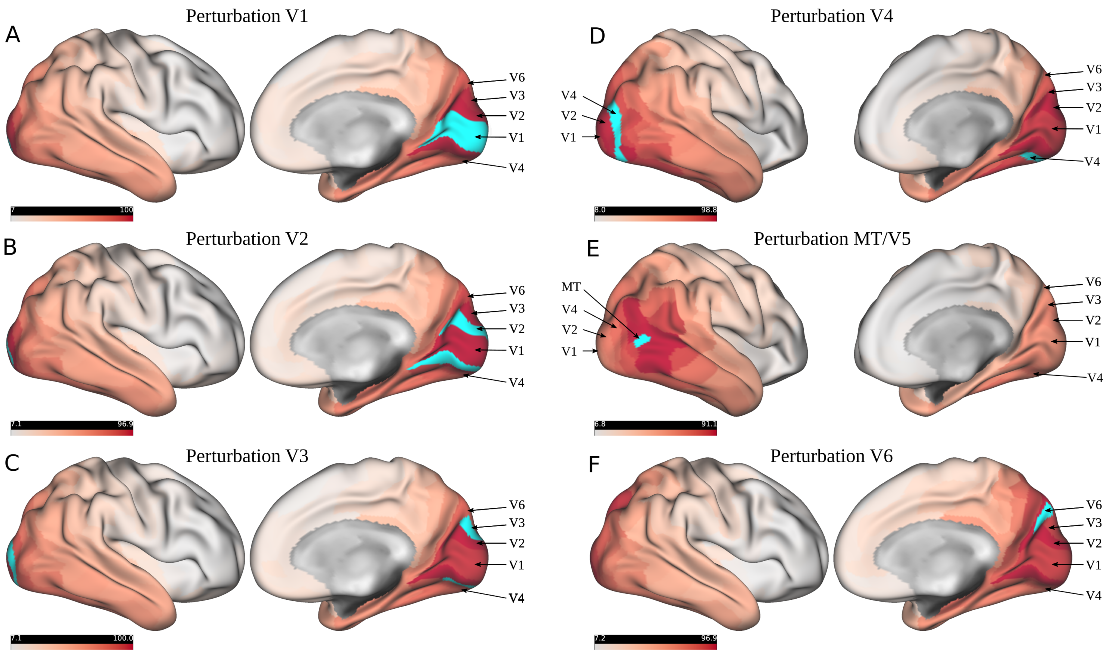

Figure S11: The figure illustrates the effect of a perturbation in a STGNN model systematically induced in target regions V1 (A), V2 (B), V3 (C), V4 (D), MT/V5 (E) and V6 (F). The connectivity values were linearly mapped between 0 and 100 and the default scaling of the color values provided by the *connectome workbench* (version 1.4.2) was used, adjusting the colormap between the $2th$ and $98th$ percentile of the values respectively.

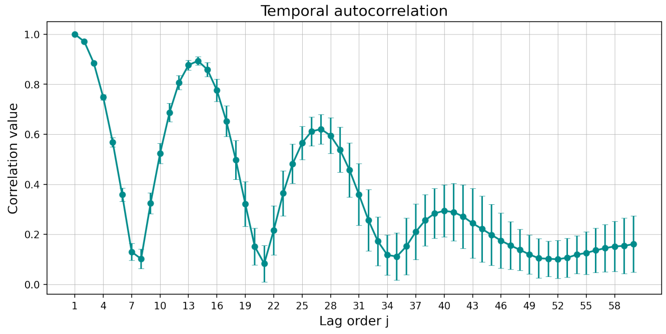

Figure S12: In this figure the temporal average autocorrelation values of fMRI timeseries in dependence of their lag order j are shown. The values were computed as the Pearson correlation between the original fMRI timeseries $\mathbf{x}_n^{(t)}$ and its lagged values $\mathbf{x}_n^{(t-j)}$, and then averaged across all subjects and brain regions. The errorbars represent the standard deviations across subjects.

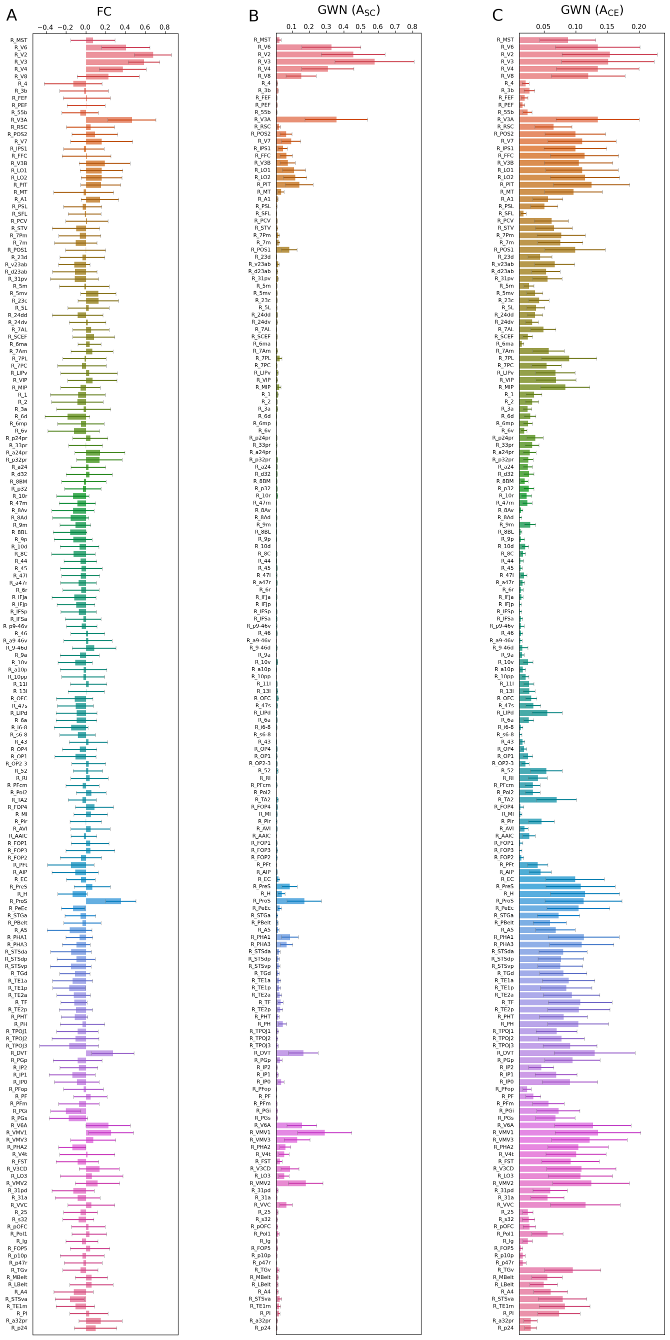

Figure S13: The FC connectivity of V1 to all other regions within the right hemisphere is shown in (A). In (B) and (C) the GWN based directed connectivity is illustrated when using the SC ($\mathbf{A}_{SC}$) and CE similarity ($\mathbf{A}_{CE}$) for the information propagation respectively.

Supplement VII

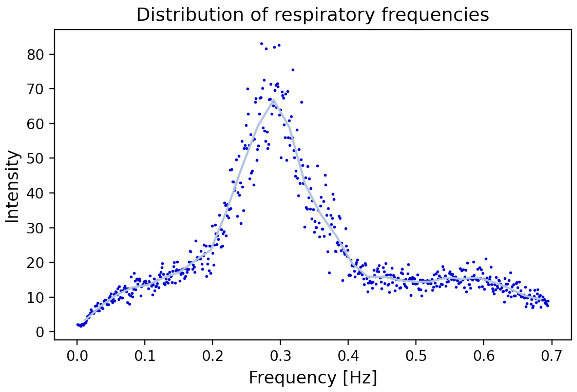

Figure S14: The distribution of respiratory frequencies is shown in this figure. For the 25 subjects resting-state fMRI dataset, physiological data was available from 22 subjects, and the depicted spectrum was computed as the average across the individual spectra of the 22 subjects.

References

[1] M. Glasser, T. Coalson, E. Robinson, C. Hacker, J. Harwell, E. Yacoub, K. Ugurbil, J. Andersson, C. Beckmann, M. Jenkinson, S. Smith, and D. Van Essen. A multi-modal parcellation of human cerebral cortex. *Nature*, 536, 2016.
